# Optimal transport for label transfer in single-cell multi-omics integration

**DOI:** 10.1093/bib/bbag334

**Published:** 2026-06-29

**Authors:** Junjun Ren, Zhengqian Zhang, Jiayu Wang, Lingyun Xie, Jialiang Wang, Meng Wang, Yongzhuang Liu

**Affiliations:** School of Computer Science and Technology, Harbin Institute of Technology, Harbin, 150001, China; School of Computer Science and Technology, Harbin Institute of Technology, Harbin, 150001, China; Department of Physics and Astronomy, UCL Centre for Data Intensive Science and Industry, University College London, London, WC1E 6BT, United Kingdom; School of Future Technology, Harbin Institute of Technology, Harbin, 150001, China; School of Computer Science and Technology, Harbin Institute of Technology, Harbin, 150001, China; Department of Respiratory Medical Oncology, Harbin Medical University Cancer Hospital, Harbin, 150081, China; School of Computer Science and Technology, Harbin Institute of Technology, Harbin, 150001, China

**Keywords:** single-cell multimodal integration, unbalanced optimal transport, label transfer, cross-modality alignment, semi-supervised learning

## Abstract

Single-cell multi-omics datasets are rapidly expanding, and integrating complementary modalities can provide a more comprehensive view of the molecular mechanisms underlying biological processes. However, cross-modality alignment remains challenging due to modality-specific measurement differences and mismatches in cell-type proportions. Here, we present single-cell Optimal Transport-based Label Transfer (scOT-LT), a semi-supervised label-transfer framework that aligns single-cell RNA sequencing (scRNA-seq) and scATAC-seq data using label-aware unbalanced optimal transport, which tolerates compositional mismatch while favoring label-consistent correspondences. scOT-LT learns a shared embedding through unbalanced optimal transport-guided alignment and transfers cell-type labels from the annotated scRNA-seq reference to unlabeled scATAC-seq via entropic OT coupling. Evaluations on multiple real-world datasets show that scOT-LT achieves strong modality mixing and high label-transfer accuracy, remains robust under downsampled scRNA-seq annotations, and can reliably detect novel cell types. Thus, scOT-LT not only improves integration and label-transfer performance but also yields explicit, interpretable cross-modality coupling, providing a practical approach for multimodal integration and annotation.

## Introduction

Rapid advances in single-cell sequencing now enable systematic investigation of cellular heterogeneity, developmental trajectories, and intercellular interactions at single-cell resolution. Emerging modalities, such as single-cell RNA sequencing (scRNA-seq) [1, 2], chromatin accessibility profiling (scATAC-seq) [3, 4], and DNA methylation profiling (e.g. snmC-seq [5] and sci-MET [6]) have significantly improved our ability to capture molecular features across multiple layers within individual cells. These technologies have deepened our understanding of cellular state transitions and provided powerful tools for dissecting the molecular mechanisms underlying complex disease [7].

Among these techniques, scRNA-seq and scATAC-seq are the most widely used for profiling transcriptomic and epigenomic information, respectively. They are highly complementary: scATAC-seq measures chromatin accessibility and highlights putative regulatory elements [8, 9], partially addressing the limited regulatory context of scRNA-seq. By contrast, scRNA-seq benefits from high-throughput data generation, mature annotation resources, and extensive reference atlases [10, 11], making it well-suited for cell-type identification and functional annotation. Joint analysis of these two modalities can therefore provide a more comprehensive view of cell states at both expression and regulatory levels, improving downstream biological inference. However, scATAC-seq data are typically sparse and noisy, which complicates accurate cell-type annotation and subsequent analyses [12]. As a result, leveraging well-annotated scRNA-seq references to transfer high-confidence labels to scATAC-seq queries has become a practical and increasingly common strategy for improving annotation quality [13].

Methodologically, single-cell multi-omics integration has evolved from purely data-driven paradigms toward approaches that explicitly incorporate biological knowledge. This shift reflects the limitations of unsupervised methods that rely only on intrinsic data structure. Classical statistical integration techniques, such as non-negative matrix factorization [14, 15] or canonical correlation analysis [16], seek shared factors or correlated features across modalities. More recent deep learning approaches, including variational autoencoder frameworks [17, 18], learn joint latent representations that can align modalities without requiring paired measurements. Besides latent-space integration methods, previous studies have also explored gene- or network-based strategies for scRNA-seq and scATAC-seq integration, where cross-modal analysis is facilitated by representing both modalities in a common gene-related feature domain or by leveraging gene-regulatory relationships between transcriptomic and epigenomic profiles [19]. Structure-aware representation learning has also been explored in single-cell analysis to better capture biologically meaningful structure [20]. While many of these integration methods are broadly applicable, approaches that rely heavily on distributional similarity or shared latent geometry can still lead to suboptimal alignment when modality gaps are large, noise levels differ substantially, or the underlying data geometry is complex. In addition, the interpretability of learned latent spaces may be limited, making it difficult to rationalize specific cross-modal correspondences.

To improve alignment accuracy and biological plausibility, semi-supervised integration methods incorporate limited prior knowledge—most commonly cell-type labels from well-annotated scRNA-seq datasets—as supervisory signals. Representative methods include scJoint [13] and scBridge [21]. scJoint jointly optimizes modality alignment and cell-type classification in a shared embedding space, enabling effective label transfer to unlabeled scATAC-seq and scaling well to atlas-level datasets. scBridge adopts an iterative curriculum. It first aligns more reliable cell populations with smaller cross-modal discrepancies and then progressively bridges more divergent populations, leveraging heterogeneity to stabilize integration. By combining supervision with geometric structure, semi-supervised methods often outperform fully unsupervised alternatives in both alignment and annotation. Nevertheless, many such methods do not yield an explicit and interpretable cell-to-cell correspondence, and their performance can still degrade under severe modality shifts or compositional imbalances.

Optimal transport (OT) provides a principled and interpretable framework for cross-modal alignment by formulating integration as the computation of a transport plan between source and target distributions [22]. Methods such as SCOT [23] compute an explicit cell-to-cell coupling matrix, enabling direct inspection of correspondences and preserving geometric relationships between modalities. However, classical OT assumes strict mass conservation, an assumption that is often violated in practice due to missing populations, batch effects, and unequal sampling across modalities. Unbalanced optimal transport (UOT) [24] relaxes this constraint by allowing partial matching via divergence-based penalties, making it more appropriate for real single-cell settings with partial overlap, noise, and imbalanced cell-type compositions. Importantly, OT frameworks can also integrate structured prior knowledge through customized cost functions, offering a natural interface between mathematical rigor and biological guidance.

In this paper, we propose single-cell Optimal Transport-based Label Transfer (scOT-LT), a semi-supervised framework that transfers cell type labels from annotated scRNA-seq reference data to unannotated scATAC-seq query data. scOT-LT employs a shared encoder to embed scRNA-seq and scATAC-seq data into a unified latent space and then aligns the two modalities by solving a UOT problem. The advantages of scOT-LT are three-fold. First, by adopting unbalanced optimal transport, scOT-LT is more robust to compositional mismatch and partial overlap between modalities. Second, its hybrid transport cost combines geometric proximity with label-consistency-based semantic constraint, leading to more biologically coherent cross-modal matching. Third, the explicit OT coupling enables interpretable cell-to-cell correspondence and supports reliable probabilistic label transfer, which is further improved by pseudo-supervised refinement with center regularization. Overall, scOT-LT provides an interpretable, coupling-driven pathway for cross-modal annotation that is robust to modality discrepancies and mismatched cell-type composition.

To evaluate the performance of scOT-LT, we compare it with six state-of-the-art methods, including scJoint [13], scBridge [21], Seurat [16], Portal [25], SCOT [23], and UnionCom [26], on four real-world datasets. The results show that scOT-LT consistently achieved strong overall performance in both paired and unpaired data integration tasks. Notably, it shows greater robustness in scenarios with incomplete reference annotations, highlighting its effectiveness in handling compositional mismatch and feature heterogeneity. In addition, scOT-LT improves detection of previously unannotated cell types in the query modality, supporting its utility for practical single-cell multi-omics label transfer.

## Materials and methods

### Data collection

We used four real-world single-cell datasets in this study, all of which are publicly available. The human SHARE-seq BMMC dataset [27] is available from the GEO database under accession GSE207308. The mouse spleen dataset [28] is available through MultiMAP at http://ngs.sanger.ac.uk/production/teichmann/MultiMAP.Thehuman multimodal PBMC dataset [29] is available from the GEO database under accession GSE156478. For the human myocardial infarction dataset [30], the gene expression matrix, gene-activity matrix, and clustering results were obtained from CellxGene at https://cellxgene.cziscience.com/collections/8191c283-0816-424b-9b61-c3e1d6258a77, and the corresponding cell-peak matrix was downloaded from Zenodo records 6578553 and 6578617.

### Overview of scOT-LT

**Figure 1:**
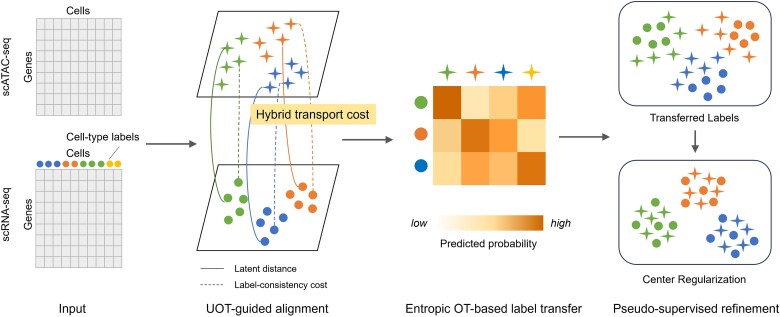
Overview of scOT-LT for cross-modal label transfer. scOT-LT integrates labeled scRNA-seq (reference) and unlabeled scATAC-seq (query) data for cross-modal label transfer. First, both modalities are embedded into a shared latent space using a shared encoder. Cross-modal alignment is learned between individual cells via unbalanced optimal transport (UOT), using a hybrid cost that combines latent geometric distance and a semantic consistency term derived from reference labels and query predictions. Next, entropic optimal transport on the aligned embedding is used to compute probabilistic label transfer from reference to query cells. Finally, the transferred labels are used for pseudo-supervised refinement with center regularization, improving cluster compactness and cell-type separability.

scOT-LT is a semi-supervised label-transfer framework for cross-modal single-cell data integration that transfers cell-type annotations from a labeled reference modality (e.g. scRNA-seq) to an unlabeled query modality (e.g. scATAC-seq), as shown in Fig. 1. The method follows a three-stage pipeline comprising cross-modality alignment, label transfer, and refinement of the integrated representation. First, cells from both modalities are projected into a shared low-dimensional embedding space. Cross-modality alignment at the cell level is achieved by optimizing an UOT objective between the reference and query embedding distributions, which improves robustness to modality-specific measurement differences and shifts in cell-type composition. The transport cost is not based solely on embedding-space distances. Instead, it combines latent-space proximity with label-consistent semantic information by incorporating reference cell-type supervision alongside query-side predictive distributions, thereby promoting alignment that is coherent in both the embedding space and the induced label space. In parallel, a cell-type classification loss guides the reference embeddings, constituting the semi-supervised component. Second, label transfer is performed via OT-based probabilistic matching in the shared embedding space, yielding a coupling matrix that encodes soft correspondences between reference and query cells. Each query cell is then assigned the label of its most probable reference match. Third, using the transferred labels as pseudo-supervision, a metric-learning refinement step further improves cross-modality mixing and cell-type separability, resulting in a more coherent joint embedding and more reliable query annotations. These components are organized as a coherent pipeline in which alignment, OT-based label transfer, and pseudo-supervised refinement are tightly connected to progressively improve the integrated representation and annotation reliability. This design enables scOT-LT to perform robust cross-modal alignment under compositional mismatch while preserving biologically meaningful correspondences and providing an interpretable basis for label transfer.

### Model

#### Problem setup and notation

We study cross-modality label transfer from a labeled reference modality (e.g. scRNA-seq) to an unlabeled query modality (e.g. scATAC-seq). For each dataset, we define a common gene set shared by the two modalities and denote its size by $M$. To obtain a shared gene representation, scATAC-seq profiles are first converted from peak-based accessibility measurements into gene-activity features using Signac [31]. This transformation aggregates accessibility signals from peaks associated with each gene, allowing the scATAC-seq data to be represented in the same gene space as the scRNA-seq expression matrix. We then retain only the genes in this shared set in both modalities, yielding matched input dimensionality before the data are passed to the shared encoder. The shared-gene representation serves to define a common input space, whereas the cross-modality alignment is performed at the cell level in the latent space via UOT with a label-consistency constraint. Let ${X}^r\in{\mathbb{R}}^{N_r\times M}$ denote the reference feature matrix with cell-type labels ${y}^r\in{\left\{1,\dots, K\right\}}^{N_r}$, and let ${X}^q\in{\mathbb{R}}^{N_q\times M}$ denote the query feature matrix, where ${N}_r$ and ${N}_q$ are the numbers of reference and query cells, respectively, and $K$ is the number of reference cell types. We denote the $i$-th reference cell by ${x}_i^r\in{\mathbb{R}}^M$ with label ${y}_i^r$, and the $j$-th query cell by ${x}_j^q\in{\mathbb{R}}^M$. During training, we sample minibatches of sizes ${B}_r$ and ${B}_q$ from the reference and query sets, respectively. Our goal is to learn a shared latent space that aligns the two modalities and enables transfer of cell-type labels from the reference to the query.

#### Model architecture

We use an encoder–classifier architecture consisting of a linear encoder and a linear classification head. The encoder ${f}_{\theta }$ maps an $M$-dimensional shared-gene feature vector $x$ to a $d$-dimensional embedding (with $d=64$),


(1) \begin{equation*} h={f}_{\theta }(x)={W}_ex+{b}_e \end{equation*}

where $h\in{\mathbb{R}}^d$ and $\theta =\left\{{W}_e,{b}_e\right\}$. A linear classifier ${g}_{\phi }$ maps the embedding $h$ to logits over the $K$ reference cell types,


(2) \begin{equation*} z={g}_{\phi }(h)={W}_ch+{b}_c \end{equation*}

where $z\in{\mathbb{R}}^K$ and $\phi =\left\{{W}_c,{b}_c\right\}$. We compute the predicted class probabilities using a softmax function,


(3) \begin{equation*} p=\mathrm{softmax}(z)\in{\left[0,1\right]}^K \end{equation*}

We denote by ${p}_i^r(k)$ the predicted probability of class $k$ for the $i$-th reference cell, and by ${p}_j^q(k)$ for the $j$-th query cell.

Overall, scOT-LT consists of three components: (i) UOT-guided cross-modality alignment, (ii) OT-based probabilistic label transfer, and (iii) pseudo-supervised refinement with center regularization.

#### UOT-guided cross-modality alignment

##### Hybrid transport cost

For a minibatch of reference embeddings ${\left\{{h}_i^r\right\}}_{i=1}^{B_r}$ with labels ${\left\{{y}_i^r\right\}}_{i=1}^{B_r}$ and query embeddings ${\left\{{h}_j^q\right\}}_{j=1}^{B_q}$, where ${h}_i^r={f}_{\theta}\left({x}_i^r\right)$ and ${h}_j^q={f}_{\theta}\left({x}_j^q\right)$, we construct a hybrid cost matrix ${C}^{UOT}\in{\mathbb{R}}^{B_r\times{B}_q}$ with entries


(4) \begin{equation*} {C}_{ij}^{UOT}=\alpha\;{C}_{ij}^{\mathrm{emb}}+{\lambda}_t\;{C}_{ij}^{\mathrm{sem}} \end{equation*}

Here, $\alpha \ge 0$ and ${\lambda}_t\ge 0$ balance embedding proximity and label consistency.

The embedding-space cost is defined as the squared Euclidean distance between embeddings,


(5) \begin{equation*} {C}_{ij}^{\mathrm{emb}}=\parallel{h}_i^r-{h}_j^q{\parallel}_2^2 \end{equation*}

The semantic cost encourages label-consistent coupling by penalizing query predictions that are incompatible with the candidate reference label. Let $e\left({y}_i^r\right)\in{\left\{0,1\right\}}^K$ be the one-hot encoding of ${y}_i^r$. With ${p}_j^q=\mathrm{softmax}\left({g}_{\phi}\left({h}_j^q\right)\right)$, we define


(6) \begin{equation*} {C}_{ij}^{\mathrm{sem}}=-\sum_{k=1}^Ke{\left({y}_i^r\right)}_k\log{p}_j^q(k)=-\log{p}_j^q\left({y}_i^r\right) \end{equation*}

Intuitively, ${C}_{ij}^{sem}$ measures how incompatible query cell $j$ is with the label of reference cell $i$. If the query classifier assigns a low probability to the reference label ${y}_i^r$, then pairing query cell $j$ with reference $i$ incurs a high semantic cost. In this way, the semantic term provides a label-compatibility measure for each reference-query cell pair. The key point is that UOT computes the transport coupling by optimizing a transport objective defined over a cost matrix. In scOT-LT, this cost matrix is given by the Eq. (4), rather than by embedding-space distance alone. Therefore, semantic label consistency is incorporated directly into the transport optimization itself, and the resulting coupling is guided jointly by latent-space geometry and label compatibility.

##### Entropically-regularized unbalanced OT alignment

We compute an unbalanced coupling $\Pi \in{\mathbb{R}}_{+}^{B_r\times{B}_q}$ by solving an entropically regularized unbalanced OT problem with relaxed marginal constraints,


(7) \begin{align*} &\underset{\Pi \ge 0}{\min}\left\langle \Pi, {C}^{UOT}\right\rangle +\mathrm{reg}\sum_{i,j}{\Pi}_{ij}\left(\log{\Pi}_{ij}-1\right)\nonumber\\&\quad+{\mathrm{reg}}_m\left(\mathrm{KL}\left(\Pi \mathbf{1}\parallel a\right)+\mathrm{KL}\left({\Pi}^{\top}\mathbf{1}\parallel b\right)\right) \end{align*}

where $\left\langle \Pi, {C}^{UOT}\right\rangle =\sum_{i,j}{\Pi}_{ij}{C}_{ij}^{UOT}$ is the matrix inner product, $\mathbf{1}$ is an all-ones vector of compatible dimension, and $\Pi \mathbf{1}\in{\mathbb{R}}^{B_r}$ and ${\Pi}^{\top}\mathbf{1}\in{\mathbb{R}}^{B_q}$ are the row and column marginals of $\Pi$. We use uniform measures $a=\frac{1}{B_r}\mathbf{1}$ and $b=\frac{1}{B_q}\mathbf{1}$. $\mathrm{KL}\left(\cdotp \parallel \cdotp \right)$ denotes the Kullback–Leibler divergence. The hyperparameters $reg$ and ${reg}_m$ control entropic smoothing and marginal relaxation, respectively.

Given $\Pi$, the unbalanced OT alignment loss is defined as the expected transport cost,


(8) \begin{equation*} {\mathcal{L}}_{UOT}=\left\langle \varPi, {C}^{UOT}\right\rangle =\sum_{i=1}^{B_r}\sum_{j=1}^{B_q}{\Pi}_{ij}{C}_{ij}^{UOT} \end{equation*}

In practice, $\Pi$ is obtained using generalized Sinkhorn iterations computed outside the automatic differentiation graph. During backpropagation, we treat $\Pi$ as a fixed coupling (stop-gradient), and propagate gradients through the cost matrix ${C}^{UOT}$.

##### Reference supervision and parameter regularization

In parallel, we supervise the classifier on labeled reference cells using cross-entropy,


(9) \begin{equation*} {\mathcal{L}}_{\mathrm{CE}}^{(r)}=-\frac{1}{B_r}\sum_{i=1}^{B_r}\log{p}_i^r\left({y}_i^r\right) \end{equation*}

where ${p}_i^r=\mathrm{softmax}\left({g}_{\phi}\left({f}_{\theta}\left({x}_i^r\right)\right)\right)\in{\left[0,1\right]}^K$ is the predicted class-probability vector for the $i$-th reference cell, and ${p}_i^r\left({y}_i^r\right)$ denotes the probability assigned to its ground-truth class ${y}_i^r$. We additionally apply an element-wise ${\mathrm{L}}_1$ penalty to the encoder and classifier parameters,


(10) \begin{equation*} {\mathcal{L}}_{reg}={\lambda}_e\parallel \theta{\parallel}_1+{\lambda}_c\parallel \phi{\parallel}_1 \end{equation*}

where $\parallel \cdotp{\parallel}_1$ denotes the sum of absolute values over all parameters, and ${\lambda}_e,{\lambda}_c\ge 0$ control the regularization strengths. The alignment stage minimizes


(11) \begin{equation*} {\mathcal{L}}_{\mathrm{align}}={\mathcal{L}}_{\mathrm{UOT}}+{\mathcal{L}}_{\mathrm{CE}}^{(r)}+{\mathcal{L}}_{\mathrm{reg}} \end{equation*}

The overall alignment-stage objective is summarized in Eq. (11), combining the UOT-based alignment loss in Eq. (8), the reference-side cross-entropy loss in Eq. (9), and the parameter regularization term in Eq. (10).

#### OT-based probabilistic label transfer

##### Entropic OT matching

After learning a shared embedding space, we transfer labels from the reference to the query using OT-based probabilistic matching. Let ${H}^r\in{\mathbb{R}}^{N_r\times 64}$ and ${H}^q\in{\mathbb{R}}^{N_q\times 64}$ denote embeddings for all reference and query cells. We construct a cost matrix ${C}^{OT}\in{\mathbb{R}}^{N_q\times{N}_r}$ using Euclidean distances in the embedding space,


(12) \begin{equation*} {C}_{ij}^{OT}=\parallel{h}_i^q-{h}_j^r{\parallel}_2 \end{equation*}

where ${C}_{ij}^{OT}$ measures the discrepancy between query cell $i$ and reference cell $j$. For numerical stability, we rescale ${C}^{OT}$to the range $\left[0,1\right]$. We then solve a balanced entropic OT problem with uniform marginals to obtain a soft coupling $\Gamma \in{\mathbb{R}}_{+}^{N_q\times{N}_r}$,


(13) \begin{equation*} \underset{\Gamma \ge 0}{\min}\left\langle \Gamma, {C}^{OT}\right\rangle +\varepsilon \sum_{i,j}{\Gamma}_{ij}\left(\log{\Gamma}_{ij}-1\right) \end{equation*}

(14) \begin{equation*} \mathrm{s}.\mathrm{t}.\Gamma \mathbf{1}=u,{\Gamma}^{\top}\mathbf{1}=v \end{equation*}

where $u=\frac{1}{N_q}{\mathbf{1}}_{N_q}\in{\mathbb{R}}^{N_q}$ and $v=\frac{1}{N_r}{\mathbf{1}}_{N_r}\in{\mathbb{R}}^{N_r}$, and $\varepsilon$ is the entropic regularization coefficient.

### Label assignment

We assign each query cell the label of its most strongly matched reference cell under $\Gamma$. Specifically, for each query cell $i$, we first identify the reference cell with the largest transport weight,


(15) \begin{align*} {j}^{\ast }=\arg \underset{j}{\max{\Gamma}_{ij}} \end{align*}

We then assign the corresponding reference label to the query cell,


(16) \begin{equation*} {\hat{y}}_i^q={y}_{j^{\ast}}^r \end{equation*}

where ${y}_j^r$ is the ground-truth label of reference cell $j$, and ${\hat{y}}_i^q$ is the pseudo-label assigned to query cell $i$. This corresponds to maximum-probability assignment under the OT coupling.

### Pseudo-supervised refinement with center regularization

To further improve within-class compactness and cross-modality separability, we perform a refinement stage that uses pseudo-labels for the query modality as additional supervision and augments the objective with a center regularizer. In this stage, we train the encoder and classifier using both labeled reference cells $\left({X}^r,\kern0.5em {y}^r\right)$ and pseudo-labeled query cells $\left({X}^q,{\hat{y}}^q\right)$, where ${\hat{y}}^q$ is obtained from the OT-based label-transfer stage.

#### Joint supervised with pseudo-labels

We supervise both modalities using cross-entropy on labeled reference cells and pseudo-labeled query cells. The combined classification loss is:


(17) \begin{equation*} {\mathcal{L}}_{CE}={\mathcal{L}}_{\mathrm{CE}}^{(r)}+{\mathcal{L}}_{\mathrm{CE}}^{(q)} \end{equation*}

(18) \begin{equation*} {\mathcal{L}}_{\mathrm{CE}}^{(q)}=-\frac{1}{B_q}\sum_{j=1}^{B_q}\log{p}_j^q\left({\hat{y}}_j^q\right) \end{equation*}

where ${p}_j^q$ is the predicted class-probability vector for the $j$-th query cell, and ${p}_j^q\left({\hat{y}}_j^q\right)$ denotes the probability assigned to its pseudo-label.

#### Center loss for class compactness

We introduce learnable class centers ${\left\{{\mu}_k\right\}}_{k=1}^K$ in the shared embedding space, where ${\mu}_y$ denotes the center associated with class $y\in \left\{1,\dots, K\right\}$. For a minibatch of embeddings $\left\{{h}_n\Big\}{}_{n=1}^B\right.$ with labels $\left\{{y}_n\Big\}{}_{n=1}^B\right.$, combining labeled reference cells and pseudo-labeled query cells, we penalize the squared Euclidean distance between each embedding and its assigned class center:


(19) \begin{equation*} {\mathcal{L}}_{\mathrm{center}}=\frac{1}{\mid B\mid}\sum_{n=1}^B\parallel{h}_n-{\mu}_{y_n}{\parallel}_2^2 \end{equation*}

where $B$ denotes the minibatch index set, and $\mid B\mid$ is its size. We compute the center loss by measuring each embedding’s distances to the center of its assigned class and averaging over the minibatch. Minimizing this loss encourages samples of the same class to concentrate around a shared prototype in the embedding space.

### Refinement objective

The refinement stage minimizes


(20) \begin{equation*} {\mathcal{L}}_{refine}={\mathcal{L}}_{CE}+{\mathcal{L}}_{\mathrm{UOT}}+\beta\;{\mathcal{L}}_{\mathrm{center}}+{\mathcal{L}}_{\mathrm{reg}} \end{equation*}

where $\beta \ge 0$ controls the weight of center regularization. Overall, the refinement-stage objective is summarized in Eq. (20), combining pseudo-supervised classification in Eq. (17), UOT-based alignment in Eq. (8), center regularization with Eq. (19), and parameter regularization in Eq. (10).

After refinement, we run OT-based matching again in the embedding space to update the query labels. If a query cell’s OT assignment differs from its previous pseudo-label, we will replace the pseudo-label with the OT assignment.

### Optimization and implementation details

To control memory usage, we compute OT costs on jointly sampled reference and query minibatches of sizes ${B}_r$ and ${B}_q$, which requires $O\left({B}_r{B}_q\right)$ pairwise cost computations per OT solve. The unbalanced formulation further improves robustness to shifts in cell-type proportions by allowing partial-mass transport. Unless otherwise specified, we optimize the encoder and classifier using SGD with momentum. When multiple dataloaders are used for the reference and query modalities, we construct matched reference-query minibatch pairs at each iteration and average the corresponding losses. Default hyperparameters, learning rate, batch size, and number of training epochs are provided in the configuration files.

## Results

### scOT-LT improves integration on paired data

To evaluate the integration and label-transfer performance, we benchmarked scOT-LT against six state-of-the-art single-cell multi-omics integration methods on a paired scRNA-seq and scATAC-seq dataset of mouse bone marrow mononuclear cells (BMMC) [32]. The pairing information was used only for evaluation, and the integration results were visualized using Uniform Manifold Approximation and Projection (UMAP). Unless otherwise stated, all quantitative results reported below are mean values over five runs with different random seeds, and statistical significance was assessed using a two-sided *t-*test.

**Figure 2:**
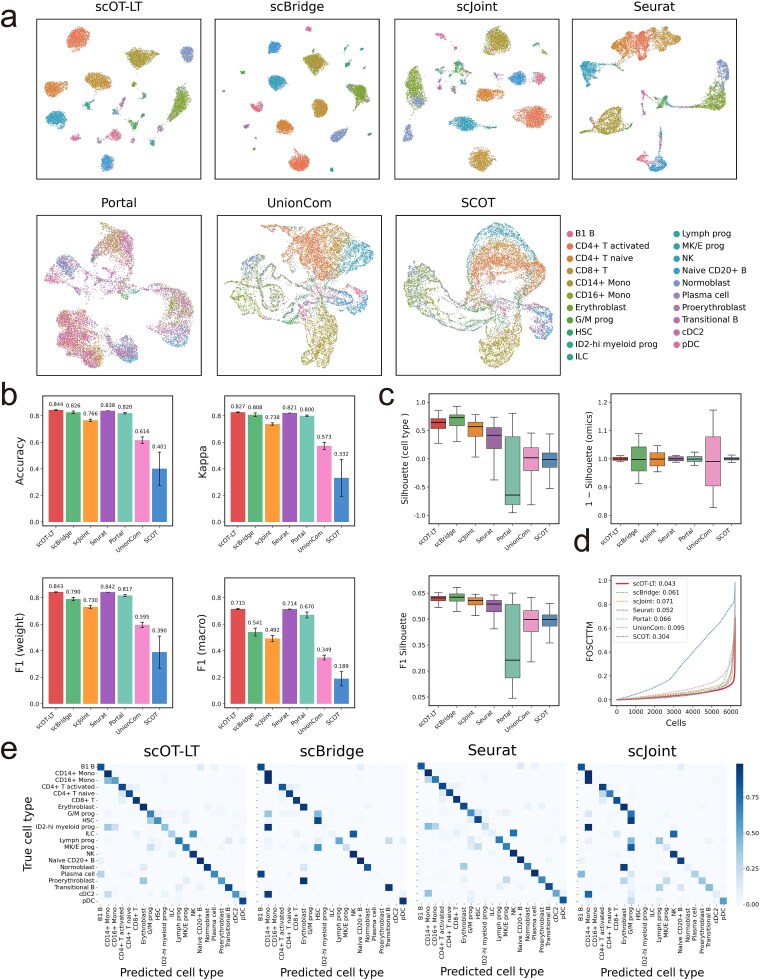
scOT-LT improves cross-modal integration and label transfer on the paired BMMC dataset. (a) UMAPs of the joint embedding for mouse bone marrow mononuclear cells (BMMC) generated by scOT-LT and baseline methods. Cells colored by cell type. (b) Summary label-transfer performance, including accuracy, Cohen’s κ, weighted F1 and macro-F1. Values are summarized over five runs with different random seeds; error bars indicate standard deviation. Statistical significance was assessed using a two-sided *t-*test. (c) Silhouette-based integration metrics quantifying preservation of cell-type structure (silhouette(cell type)), removal of modality effects (1 − silhouette(omics)), and their combined trade-off (F1 silhouette). (d) Cross-modality alignment accuracy measured by FOSCTTM; lower values indicate more accurate cell-to-cell correspondence. (e) Confusion matrices of transferred labels (rows: True cell type; columns: Predicted cell type).

The UMAP plots show that scOT-LT preserves clear cell-type separation while effectively mixing scRNA-seq and scATAC-seq cells within each cluster (Fig. 2a and Supplementary Fig. S1a). In contrast, several baselines, such as Portal, UnionCom, and SCOT, show either poorer cross-modality mixing or weaker preservation of biological structure in the embedding, consistent with their inferior quantitative performance. Quantitatively, scOT-LT attains near-optimal modality mixing (1 − Silhouette(omics) = 1.000; Fig. 2c) and achieves the highest F1 silhouette coefficient (0.602), indicating the most favorable trade-off between removing modality-specific effects and maintaining biological structure among the compared methods. Although scBridge shows a slightly higher cell-type silhouette coefficient (0.522) than scOT-LT (0.515), this difference is not statistically significant (*t-*test *P*-value = 0.524), whereas scBridge shows clearly weaker label-transfer performance, most notably with a substantially lower macro-F1 (0.541 versus 0.715; *t-*test *P*-value = 1.47e−4). This suggests that improved cluster separation alone does not necessarily translate into more reliable cross-modality annotation, especially for low-abundance cell populations (Fig. 2b and 2e). Seurat achieves comparable modality mixing but weaker cell-type separation (cell-type silhouette = 0.304) and a lower F1 silhouette (0.566), indicating that its embedding sacrifices biological granularity despite good overall mixing (Fig. 2c).

Consistent with the summary label-transfer metrics (Fig. 2b), scOT-LT achieves the strongest overall label-transfer performance on the BMMC dataset, with the highest accuracy (0.844), weighted F1 (0.843), macro-F1 (0.715), and Cohen’s kappa (0.827). Compared with scJoint, scBridge, Portal, SCOT, and UnionCom, the improvements in label-transfer performance are all statistically significant (*t-*test *p*-value<0.05). Relative to Seurat, scOT-LT also shows significantly higher accuracy and kappa, while the differences in weighted F1 and macro-F1 are not statistically significant. Notably, macro-F1 is particularly informative here because the BMMC dataset is highly imbalanced, with many rare populations containing fewer than 100 cells (e.g., cDC2: 36, HSC: 41, Lymph prog: 52, Plasma cell: 59, MK/E prog: 64, CD16+ Mono: 66, and ID2-hi myeloid prog: 67). The confusion matrices (Fig. 2e and Supplementary Fig. S1b) indicate that scBridge and scJoint tend to collapse closely related myeloid subtypes into majority-class labels, such as mapping CD16+ Mono and cDC2 to CD14+ Mono, which can keep accuracy and weighted F1 relatively high while substantially reducing recall for rare cell types. By contrast, scOT-LT better preserves fine-grained identities and improves recovery of low-abundance populations. Seurat performs strongly across major lineages and achieves competitive overall scores with an accuracy of 0.838 and a macro-F1 of 0.714, but still struggles with difficult, rare subsets such as ILC and ID2-hi myeloid progenitors, which scOT-LT better recovers. Moreover, Supplementary Fig. S1b highlights consistent B-lineage confusion in SCOT and UnionCom (e.g. B1 B cells are frequently misclassified as Naive CD20+ B cells), whereas scOT-LT maintains higher B-cell subtype fidelity. Furthermore, scOT-LT yields the lowest FOSCTTM (0.044; Fig. 2d), indicating lower alignment error than the competing methods, with statistically significant improvements over scBridge, UnionCom, SCOT, and Seurat (*t-*test *P*-value<0.001). Together, these observations suggest that scOT-LT’s label-transfer improvements largely stem from better preservation of rare and closely related cell types, as reflected by its stronger macro-F1, lower alignment error, and more diagonal-dominant confusion patterns.

In addition, to further illustrate the interpretability of the learned OT coupling, we visualized a representative submatrix of the coupling matrix that encodes cross-modal cell-to-cell correspondences. As shown in Supplementary Fig. S2, the coupling exhibits a clear block-wise structure when scATAC-seq and scRNA-seq cells are grouped by their annotated cell types. This pattern provides an interpretable cell-level view of the learned correspondences and supports that the OT-based alignment preserves cell-type-consistent cross-modal matching.

### scOT-LT improves integration when cross-modality correspondence is unpaired

We further evaluated scOT-LT in an unpaired cross-modality integration setting using a mouse spleen dataset comprising 4333 scRNA-seq cells and 3166 scATAC-seq cells [28], where cells are not paired across modalities and cross-modality correspondence must be inferred from shared distributional structure.

**Figure 3:**
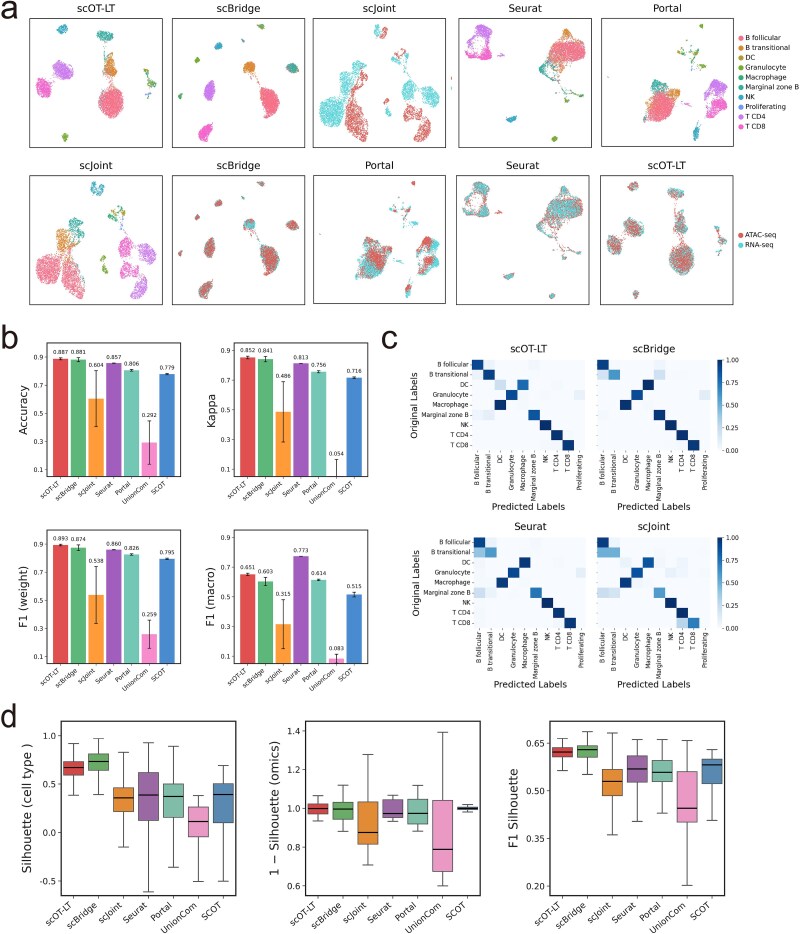
scOT-LT improves unpaired cross-modality integration and label transfer in the mouse spleen dataset. (a) Joint UMAP embeddings of scRNA-seq and scATAC-seq cells generated by scOT-LT and competing methods. Top row: Cells colored by cell type. Bottom row: Cells colored by modality. (b) Summary label-transfer performance, including accuracy, Cohen’s κ, weighted F1 and macro-F1. Values are summarized over five runs with different random seeds; error bars indicate standard deviation. Statistical significance was assessed using a two-sided *t-*test. (c) Confusion matrices comparing true and predicted labels across methods. (d) Silhouette-based integration metrics quantifying preservation of cell-type structure (silhouette(cell type)), removal of modality effects (1 − silhouette(omics)), and their combined trade-off (F1 silhouette).

In the joint UMAP embedding, scOT-LT preserves cell-type structure while improving cross-modality alignment (Fig. 3a and Supplementary Fig. S3a). When colored by cell type, scOT-LT forms compact, well-separated clusters with clear boundaries between major immune populations. When colored by modality, scRNA-seq and scATAC-seq cells substantially overlap within the same cell-type regions, suggesting that the embedding is driven primarily by biological identity rather than modality-specific variation. In contrast, scJoint, Portal, and SCOT show weaker separation between cell-type clusters and reduced cluster compactness, whereas UnionCom yields a more diffuse embedding with increased within-cell-type dispersion and reduced cluster cohesion. Consistent with this, scJoint and UnionCom exhibit modality-driven organization, with partial segregation of RNA and ATAC cells. These observations are supported by silhouette-based embedding metrics (Fig. 3d). scOT-LT achieves a strong cell-type silhouette (0.626) and near-optimal modality mixing (1 − Silhouette(omics) = 1.000), while attaining the highest F1 silhouette (0.619), indicating a favorable balance between structural preservation and cross-modality mixing. Although scBridge shows a slightly higher cell-type silhouette (0.630), the difference is not statistically significant (*t-*test *P*-value = 0.740), and scOT-LT still shows clearly delineated cluster boundaries in the UMAP embedding.

We next evaluated cross-modality label-transfer performance (Fig. 3b). scOT-LT achieves the highest accuracy (0.887), weighted F1 (0.893), and Cohen’s kappa (0.852) among all methods. Its macro-F1 (0.651) is second only to Seurat (0.773) and remains substantially higher than other baselines (*t-*test *P* < 0.05), indicating that scOT-LT improves overall correctness without relying solely on majority classes. Confusion matrices further substantiate these results (Fig. 3c and Supplementary Fig. S3b). Notably, scBridge, scJoint, and Seurat show pronounced errors on B transitional cells (constituting 7% of scATAC-seq), whereas scOT-LT achieves substantially higher label transfer for this population. Additionally, most benchmark methods struggle with DC cells (constituting 2% of scATAC-seq). In particular, scBridge, scJoint, and Seurat misassign nearly all DC cells to Macrophages, whereas scOT-LT markedly reduces this confusion.

Collectively, these results demonstrate that scOT-LT supports reliable unpaired integration, achieving strong label transfer while maintaining high modality mixing and clear cell-type separation, including improved annotation of low-frequency populations.

### scOT-LT is robust to incomplete scRNA-seq annotations

Supervised and semi-supervised integration methods can be sensitive to the completeness of input cell-type annotations. In practical applications, however, scRNA-seq labels are frequently incomplete, with a non-trivial fraction of cells remaining unlabeled because of ambiguous marker expression or limited reference coverage. To evaluate robustness under incomplete labels, we performed controlled label downsampling on the unpaired mouse spleen dataset by randomly retaining p% of scRNA-seq cell-type labels [28], with $p\in \left\{0.2,0.5,0.8,1.0\right\}$. For each $p$, we repeated the procedure five times and assessed performance using label-transfer metrics (accuracy, Cohen’s $\kappa$, weighted F1, and macro-F1) together with embedding-quality metrics (cell-type silhouette, $1-$Silhouette(omics), and F1 silhouette).

**Figure 4:**
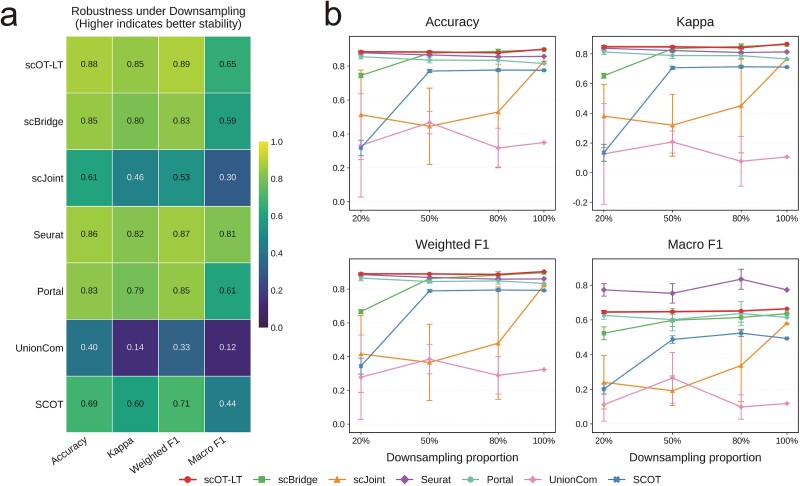
Robustness of scOT-LT to incomplete scRNA-seq annotations. (a) Robustness scores of label-transfer performance under controlled downsampling of scRNA-seq annotations. Robustness is quantified as the normalized area under the performance curve (AUC) over label-retention rates p∈{0.2,0.5,0.8,1.0}, with higher values indicating greater robustness. Metrics include accuracy, Cohen’s κ, weighted F1, and macro-F1. (b) Label-transfer performance as a function of annotation completeness. Line plots show mean accuracy, Cohen’s κ, weighted F1, and macro-F1 across five random repeats at each downsampling level. Error bars indicate standard deviation. Statistical significance was assessed using a two-sided *t-*test.

Across downsampling levels, scOT-LT exhibits consistently stable label-transfer performance (Fig. 4b). As supervision decreases, accuracy, Cohen’s $\kappa$, and weighted F1 remain high with only limited variation; even when only 20% of labels are retained, performance does not show a pronounced decline, suggesting that scOT-LT does not rely heavily on dense label supervision to learn cross-modality alignment. In contrast, scBridge and SCOT show clearer performance degradation at $P=20\%$, suggesting that a higher degree of supervision is needed for reliable matching. scJoint and UnionCom display more volatile and sometimes non-monotonic trends, which may reflect sensitivity to which labeled cells are retained in each repeat, especially when label coverage for minority populations becomes discontinuous. Macro-F1 varies more across methods than weighted F1, consistent with its greater sensitivity to minority-class performance. Notably, Seurat maintains the most favorable macro-F1 trend across $p$, while scOT-LT remains competitive rather than collapsing under sparse supervision. Embedding quality curves show a similar pattern (Supplementary Fig. S4b). scOT-LT preserves cell-type structure across $p$, maintaining stable cell-type silhouette and F1 silhouette, whereas scJoint and UnionCom exhibit weaker or less stable structure preservation. Meanwhile, modality-mixing score is close to the ceiling for several approaches and varies only slightly with label downsampling, indicating limited discriminative power in the high-mixing regime.

To summarize robustness, we quantified stability as the normalized area under the performance curve (AUC) over $p\in \left[\mathrm{0.2,1.0}\right]$, which captures both the absolute performance and its stability across varying levels of supervision. scOT-LT achieves the highest AUC for most label-transfer and embedding-quality metrics, including accuracy (0.884), Cohen’s $\kappa$ (0.848), and weighted F1 (0.890), cell-type silhouette (0.600), $1-$Silhouette(omics) (0.997), and F1 silhouette (0.614) (Fig. 4a and Supplementary Fig. S4a). These advantages are statistically significant for the main label-transfer metrics relative to most competing methods. Macro-F1 shows a more nuanced pattern. Although scOT-LT (0.650) significantly outperforms most baselines (*t-*test *P*-value<0.05), it remains lower than Seurat (0.785). For embedding quality, scOT-LT also achieves the strongest overall performance, with significant gains in cell-type silhouette and F1 silhouette, while modality-mixing score varies only slightly across methods, indicating limited discriminative power in the high-mixing regime. Together, these observations suggest that scOT-LT remains robust under incomplete reference annotations, maintaining strong overall label transfer performance and embedding quality even when supervision is substantially reduced.

### scOT-LT enables reliable detection of novel cell populations

In many label-transfer settings, the reference data may not fully cover all cell types in the query dataset. To evaluate scOT-LT under this condition, we used a multimodal peripheral blood mononuclear cell (PBMC) dataset comprising 4644 CITE-seq cells and 4502 ASAP-seq cells, annotated into seven and nine cell types, respectively [29]. Seven cell types are shared across modalities, whereas two cell types occur only in ASAP-seq and are absent from the CITE-seq reference. These ASAP-seq-specific cell types were defined according to the original dataset annotations. Specifically, cell populations annotated in ASAP-seq but absent from the CITE-seq reference labels were treated as ground-truth novel cell types for evaluation.

**Figure 5:**
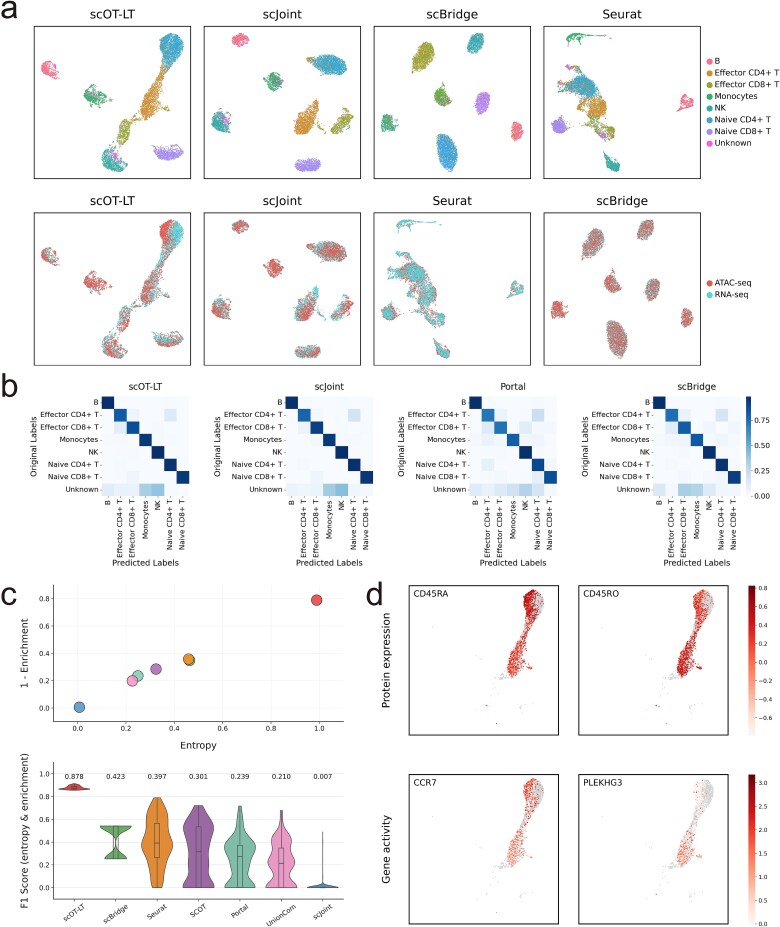
scOT-LT detects query-specific cell types in the multimodal PBMC CITE-seq and ASAP-seq dataset. (a) Joint UMAP embeddings generated by scOT-LT and competing methods. Top row: Cells colored by cell type. Bottom row: Cells colored by modality. (b) Confusion matrices comparing true and predicted labels across methods. (c) Quantification of uncertainty for ATAC-specific novel cell types using normalized entropy, enrichment, and their combined F1 score. Methods that avoid over-assignment of novel cells to a single reference label exhibit higher entropy and lower enrichment. (d) Protein expression of CD45RA and CD45RO and gene-activity signals of CCR7 and PLEKHG3 projected onto the scOT-LT UMAP of effector CD4+ T and naive CD4+ T cells.

In the joint embedding, scOT-LT produces distinct cell-type clusters with substantial interleaving of scRNA-seq and scATAC-seq cells within each cluster (Fig. 5a and Supplementary Fig. S5a), achieving strong embedding quality across cell-type separation (cell-type silhouette = 0.549), modality mixing (1 − Silhouette(omics) = 0.977), and overall F1 silhouette (0.599) (Supplementary Fig. S6c). On the seven shared cell types, scOT-LT achieves strong overall label-transfer performance (Fig. S6c), with an accuracy of 0.927, Cohen’s κ of 0.912, a weighted F1 score of 0.927, and a macro-F1 score of 0.935. scJoint is the only method with comparable mean scores, but the differences between scOT-LT and scJoint are not statistically significant for these shared-type label-transfer metrics. By contrast, scOT-LT significantly outperforms scBridge, Portal, SCOT, UnionCom, and Seurat on all four shared-type metrics (*t-*test *P*-value<0.001). Confusion matrices further support these performance patterns, showing that baseline errors are mainly concentrated in confusions between closely related T-cell states, whereas scOT-LT maintains a more diagonal label-transfer structure (Fig. 5b and Supplementary Fig. S6b).

For scATAC-seq-specific cell types, correct assignment to the reference label set is inherently impossible because the corresponding labels are absent from the scRNA-seq reference. In this setting, an ideal model express uncertainly through a diffuse probability distribution over known labels rather than forcing assignment to a single reference category. We quantified the novel type of uncertainty behavior using entropy, enrichment, and their harmonic-mean F1 score. As shown in Fig. 5c, scOT-LT exhibits the most favorable uncertainty profile on ATAC-specific cells, with the highest entropy (0.988), the lowest enrichment (0.212), and the highest novel-type F1 score (0.877). Importantly, the novel-type F1 of scOT-LT is significantly higher than that of all competing methods (*t-*test*P*-value<0.01). Supplementary Fig. S5b further shows that ATAC-specific cells are dispersed across multiple regions of the scOT-LT embedding rather than concentrated in common clusters, consistent with the model expressing uncertainty for these cells. In contrast, scJoint represents a common over-assignment failure mode, with extremely low entropy (0.005), near-maximal enrichment (0.996), and a novel-type F1 score of 0.005 (Fig. 5c), while other baselines show only intermediate uncertainty behavior.

We further examined marker concordance in the PBMC dataset as a biological validation of the learned representation. Specifically, CD45RA protein and CCR7 gene activity, which are characteristic of naive CD4+ T cells, were enriched in one region of the embedding, whereas CD45RO protein and PLEKHG3 gene activity, associated with effector-like CD4+ T cells, were enriched toward another region (Fig. 5d). These concordant protein- and gene-activity patterns indicate that the integrated representation captures a biologically meaningful continuum between naive-like and effector-like CD4+ T-cell states.

Finally, we performed an ablation on the PBMC dataset without protein measurements, using only gene expression and gene-activity features. scOT-LT produced a coherent joint embedding and meaningful cross-modal label transfer, although performance was weaker than in the full multimodal setting (Supplementary Fig. S7). This result suggests that protein measurements are beneficial for improving alignment and annotation quality.

Taken together, these results demonstrate that scOT-LT not only achieves strong cross-modality label transfer for shared cell types but also reliably avoids systematic over-assignment when the reference atlas is incomplete, providing a practical advantage for real-world multimodal integration scenarios in which unseen or modality-specific populations are common.

### Practical scalability of scOT-LT on a large-scale dataset

**Figure 6:**
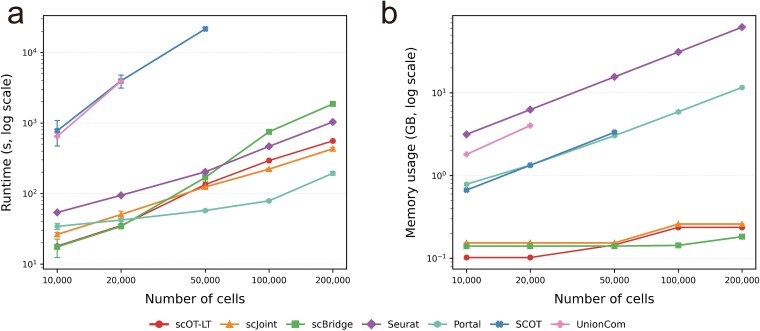
Runtime and memory usage of scOT-LT and baseline methods across increasing dataset sizes. Runtime (a) and memory usage (b) are shown on a logarithmic scale. Each point represents the mean over five independent runs on randomly subsampled datasets at each cell count. Methods that failed to complete or exceeded memory limits at larger scales are omitted for those conditions.

To further assess the practical scalability of scOT-LT, we evaluated it on a substantially larger human myocardial infarction dataset containing 191,795 scRNA-seq cells and 46,086 scATAC-seq cells [30]. We constructed subsets of increasing size (10k, 20k, 50k, 100k, and 200k cells) and measured runtime, memory usage, and predictive performance. As the number of cells increased, the runtime of scOT-LT rose in a controlled manner (Fig. 6a), while remaining substantially lower than that of several computationally intensive baselines at comparable scales. Meanwhile, the memory usage of scOT-LT stayed low across all tested scales and was markedly lower than that of more memory-demanding methods such as Seurat and Portal (Fig. 6b).

Importantly, the predictive performance of scOT-LT remained stable as dataset size increased (Supplementary Fig. S8). The accuracy remained around 0.90–0.92, and the macro-F1 remained around 0.76–0.78 across the tested scales, without noticeable degradation on larger subsets. These results suggest that scOT-LT can be applied to substantially larger datasets while maintaining manageable computational cost and stable annotation performance.

## Discussion

scOT-LT provides a unified framework for cross-modal single-cell integration and label transfer. From a methodological perspective, its main strengths are the integration of robustness, interpretability, and supervision within a single OT-based framework. In particular, scOT-LT combines UOT for improved tolerance to partial overlap and compositional mismatch, a hybrid cost for label-consistent matching, and an explicit coupling formulation that makes the resulting label transfer more interpretable. Across the evaluated settings, scOT-LT achieves strong performance in both label-transfer accuracy and embedding quality, while showing appropriate behavior when the query contains cell populations absent from the reference.

A key feature of scOT-LT is its use of UOT for cross-modality alignment. Unlike balanced OT, which implicitly assumes comparable cell-type compositions across modalities, UOT relaxes the marginal constraints and allows partial-mass transport, making the alignment less sensitive to compositional mismatch between the reference and query. This robustness is particularly relevant for multimodal data, where differences in capture efficiency, feature sparsity, or preprocessing can shift apparent population proportions. In addition, scOT-LT uses a hybrid transport cost that is not determined solely by distances in the embedding space. By incorporating reference supervision alongside query-side predictive distributions, the transport objective discourages label-inconsistent couplings and promotes correspondences that are coherent across both the learned representation space and the induced label space. This design helps explain why scOT-LT maintains a favorable balance between modality mixing and cell-type separation in the joint embedding.

On shared cell types, scOT-LT consistently yields strong label transfer and reduces common error patterns observed in baseline methods. Importantly, these gains are not achieved by collapsing structure in the integrated space. Embedding metrics indicate that scOT-LT preserves cell-type geometry while achieving near-ceiling mixing across modalities. In contrast, some approaches display weaker cluster separation or more fragmented embedding regions, reflecting less stable cross-modality alignment or overreliance on geometric proximity alone.

Beyond shared populations, scOT-LT explicitly addresses the realistic case in which the query contains cell types absent from the reference. In this setting, assigning a confident label to a known reference label is inherently incorrect. scOT-LT, in contrast, tends to produce diffuse predictive distributions over known labels for query-specific cells, which can be quantified using entropy- and enrichment-based uncertainty metrics. This uncertainty-aware behavior provides a practical mechanism to prioritize candidate novel populations for manual inspection and secondary annotation, rather than silently absorbing them into nearby reference classes. By contrast, methods that rely heavily on supervised classification or rigid alignment can over-assign novel cells to known categories, yielding overly confident but misleading annotations.

The pseudo-supervised refinement stage further stabilizes the joint representation by training on labeled reference cells together with pseudo-labeled query cells, while center regularization improves within-class compactness. Reusing the OT-based alignment objective during this stage helps maintain consistency between initial alignment and subsequent representation refinement. Another practically important property of scOT-LT is its robustness to sparse supervision. The label-downsampling experiments show that the method maintains strong overall performance even when only a fraction of reference labels is retained, suggesting that its alignment mechanism does not depend excessively on dense annotation. This robustness is particularly relevant in realistic settings where reference labels may be incomplete, noisy, or unevenly distributed across cell populations.

In addition to its predictive performance, the current implementation of scOT-LT was designed with practical scalability in mind. During training, OT is computed on jointly sampled reference and query mini-batches, so the cost of each OT solve depends on batch size rather than the total number of cells in the dataset. Moreover, the backbone model is lightweight, consisting of a linear encoder and a linear classifier that operate in a low-dimensional embedding space. These design choices make the optimization substantially more memory-efficient than full-dataset OT coupling and help explain why scOT-LT remains practically applicable on larger datasets, as also supported by the scalability experiments.

Several promising directions remain for future work to extend scOT-LT. The current implementation uses a shared gene set and gene-activity conversion as a practical bridge for RNA–ATAC integration, but this should be viewed as one instance of a broader shared-representation strategy rather than a restriction of the framework itself. More generally, extending scOT-LT to other multi-omics combinations, including transcriptome-proteome settings, will likely require more suitable bridge representations or modality-specific encoders, especially when the directly shared feature space between modalities is limited. In addition, scOT-LT is formulated for semi-supervised cross-modality annotation with a labeled reference. Although our downsampling experiments indicate robustness to sparse supervision, the current framework is not designed for the fully unlabeled setting, which would require replacing label-aware components with unsupervised or self-supervised objectives. Extending the framework to fully unlabeled multimodal alignment is an important direction for future work. Finally, while uncertainty can highlight query-specific populations, downstream workflows for validating and characterizing these candidate novel types warrant further development.

**Key Points**

- We propose scOT-LT, a semi-supervised framework for cross-modal single-cell integration and label transfer from scRNA-seq to scATAC-seq.
- scOT-LT combines unbalanced optimal transport (UOT) with a hybrid transport cost that integrates latent geometric proximity and label-consistency supervision, enabling robust cell-level alignment under modality discrepancy and mismatched cell-type composition.
- The OT formulation provides an explicit and interpretable coupling between modalities, which directly supports probabilistic label transfer and pseudo-supervised refinement.
- scOT-LT outperforms state-of-the-art methods in integration quality and label transfer, remains robust to incomplete reference annotations, and supports reliable detection of query-specific (novel) cell populations.

## Supplementary Material

Supplementary_Information_bbag334

## Data Availability

scOT-LT was implemented in PyTorch, and the code is available at https://github.com/yongzhuangliulab/scOT-LT.
