# Supplementary material for: Optimal transport for label transfer in single-cell multi-omics integration
Source: Brief Bioinform. 2026 Jun 29;27(3):bbag334. doi: 10.1093/bib/bbag334 (PMC13313528; doi:10.1093/bib/bbag334)
Supplement: Supplementary_Information_bbag334 [file supplementary_information_bbag334.docx]

SUPPLEMENTARY INFORMATION

Optimal transport for label transfer in single-cell multi-omics integration

Supplementary Figures ------------2-9

Supplementary Notes ------------10-17

Reference ------------18


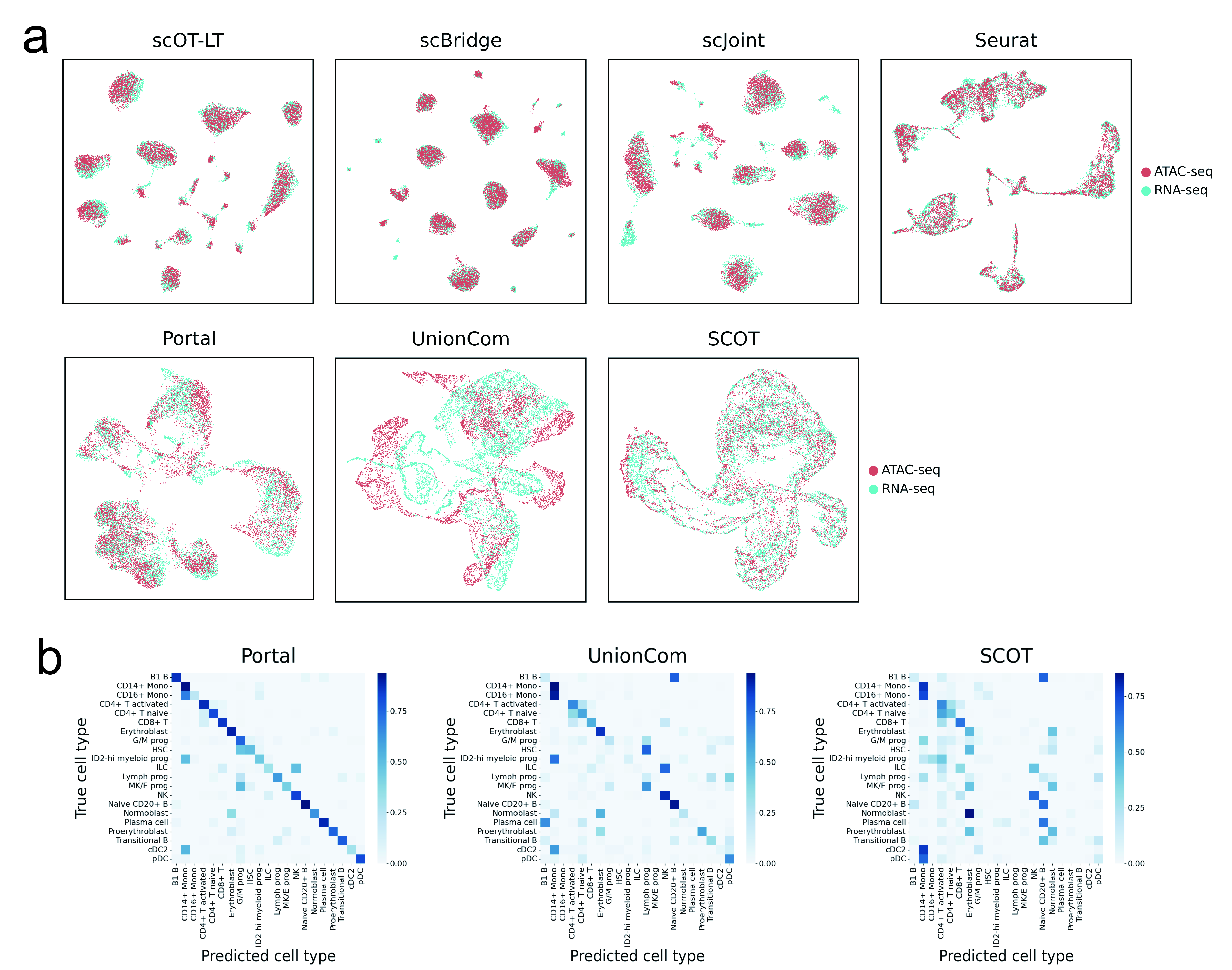


**Supplementary Figure S1.** scOT-LT and baseline performance on paired BMMC multi-omics integration and label transfer. **(a)** UMAP embeddings of the paired mouse bone marrow mononuclear cell (BMMC) scRNA-seq and scATAC-seq data. Cells are colored by modality. **(b)** Confusion matrices for label transfer from scRNA-seq (reference) to scATAC-seq (query) for Portal, UnionCom and SCOT. Rows indicate true cell types and columns indicate predicted labels; color intensity denotes the fraction of query cells assigned to each reference label.


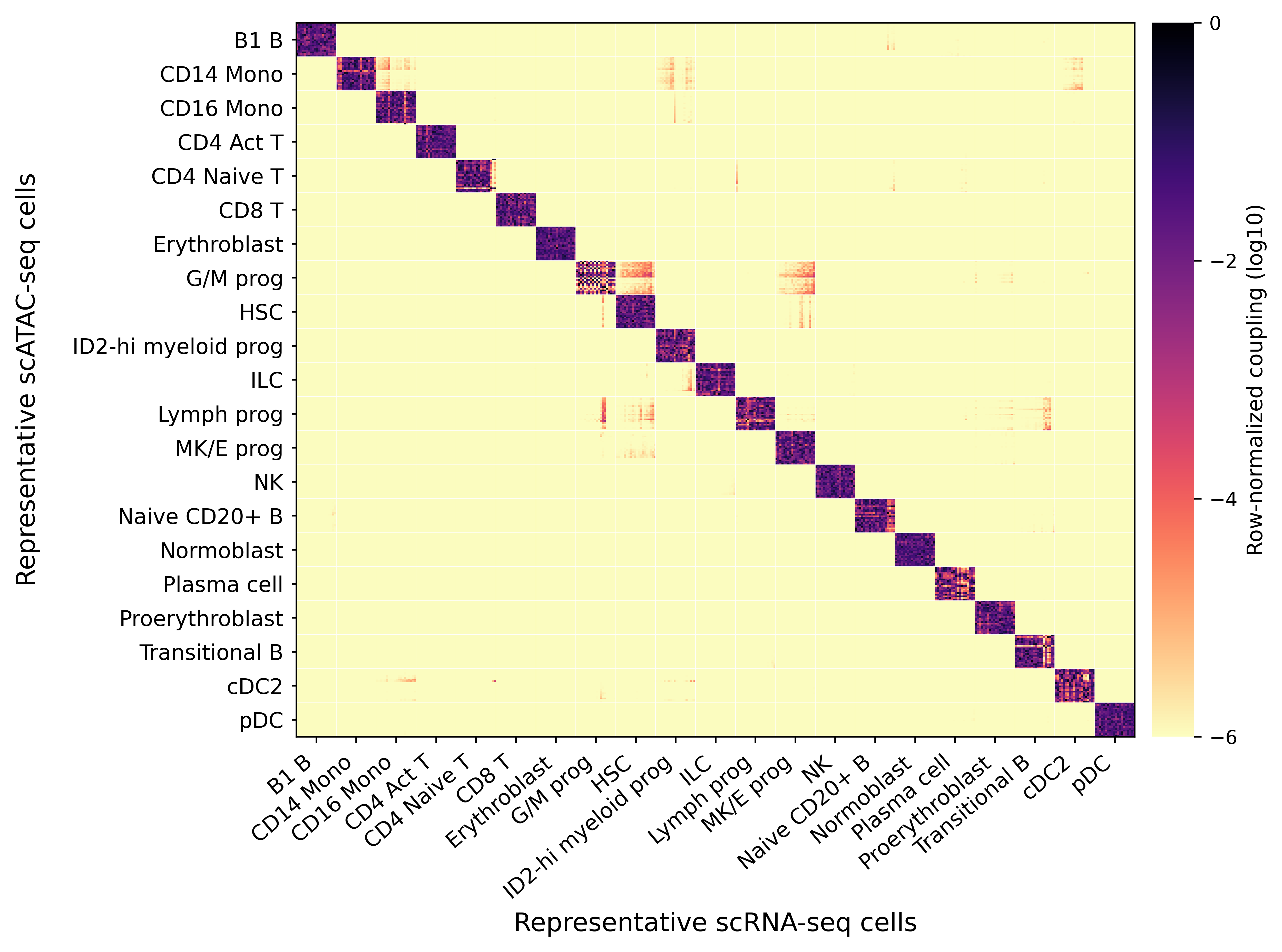


**Supplementary Figure S2.** Visualization of the learned OT coupling matrix for representative cell subsets. Heatmap of a representative submatrix of the OT coupling matrix between scATAC-seq cells (rows) and scRNA-seq cells (columns), with both axes ordered by annotated cell type. For visualization, coupling values are row-normalized and log-transformed. The block-wise structure indicates that transport mass is concentrated on cell-type-consistent cross-modal matches.


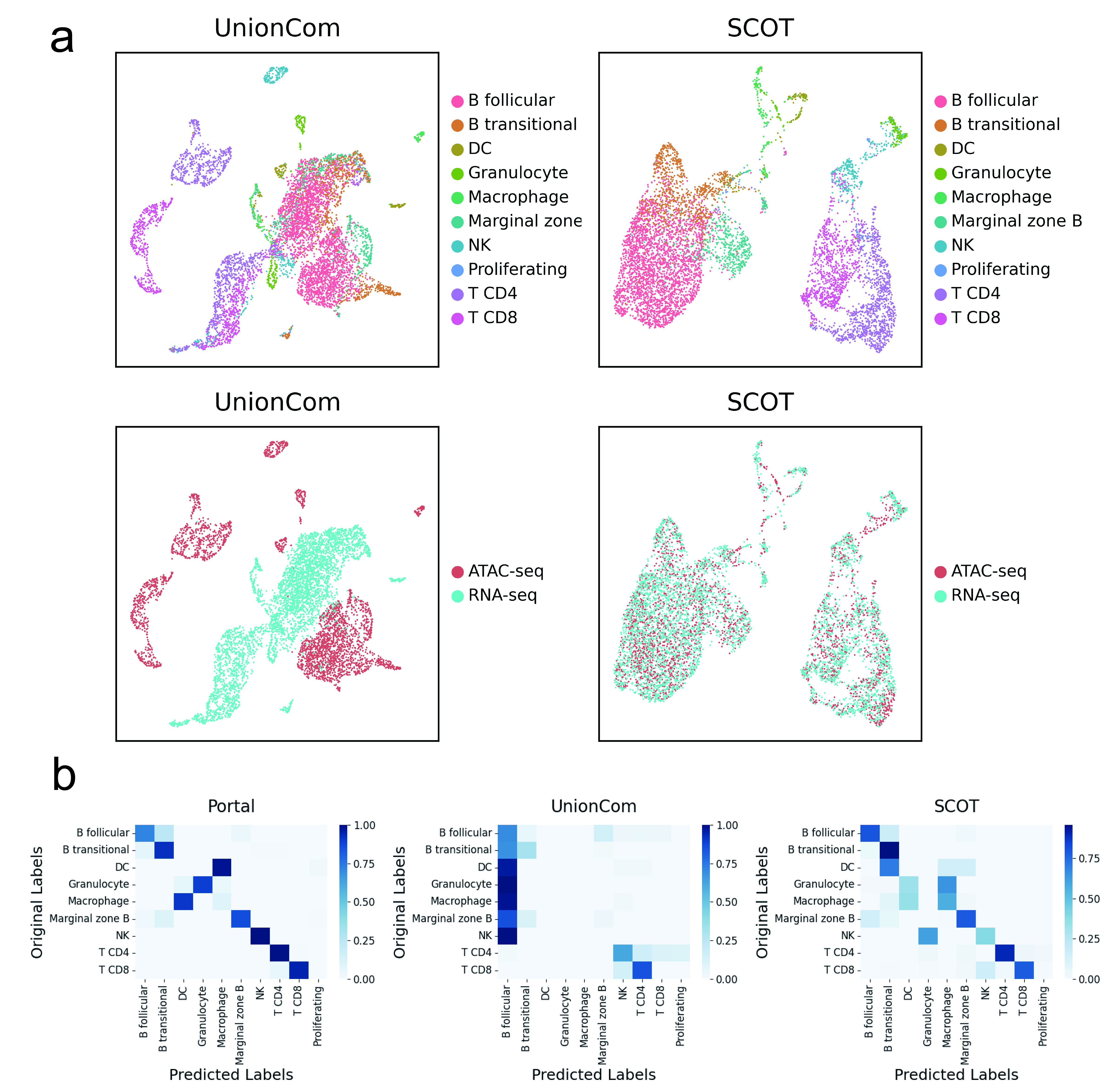


**Supplementary Figure S3.** Additional visualization and label transfer evaluation in unpaired mouse spleen integration. **(a)** UMAP embeddings produced by SCOT, and UnionCom in the unpaired spleen dataset. Cells are colored by cell type (top) and modality (bottom). **(b)** Confusion matrices summarizing label transfer performance for Portal, SCOT, and UnionCom.


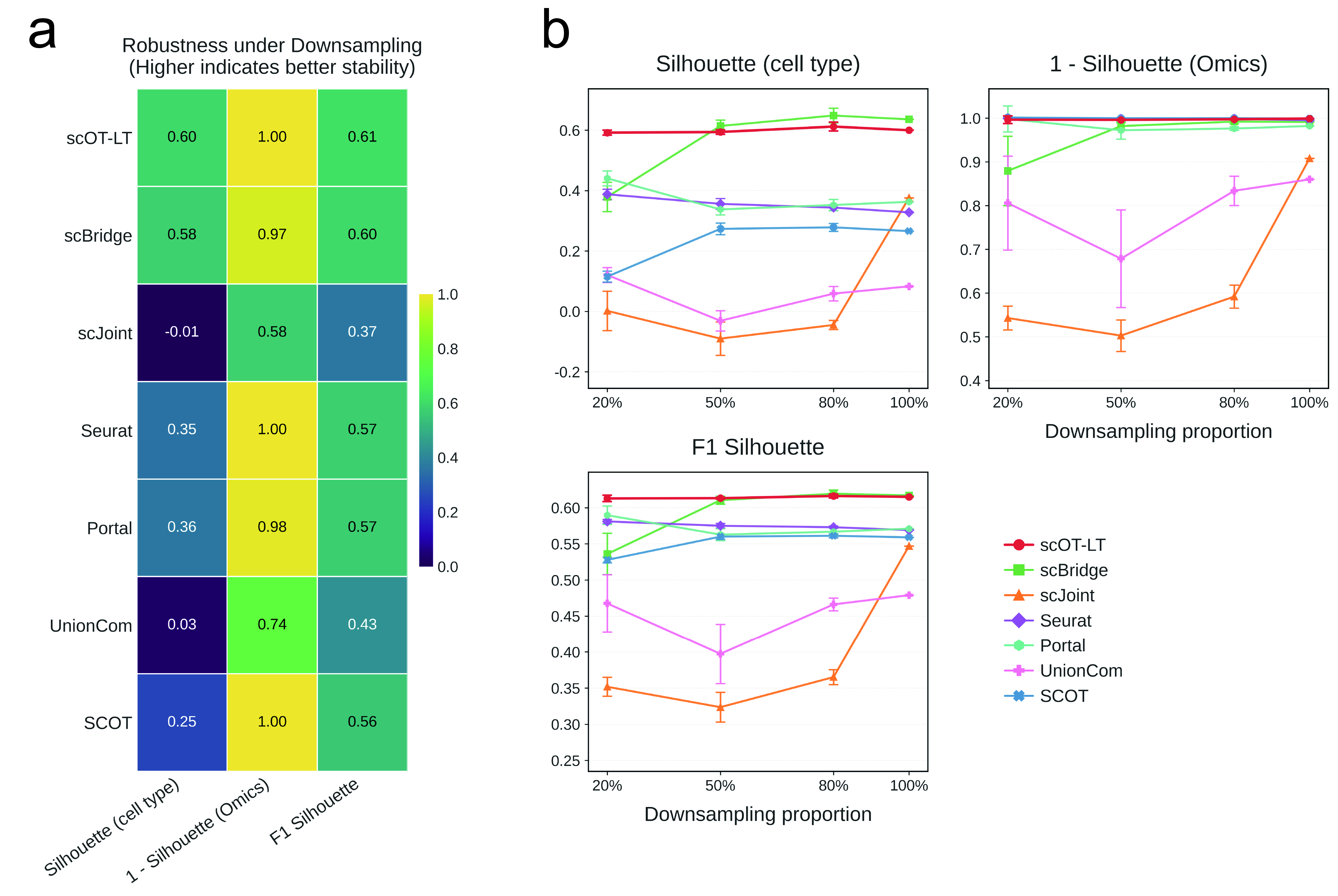


**Supplementary Figure S4.** Stability of embedding quality under label downsampling. **(a)** Robustness scores of embedding quality metrics under scRNA-seq label downsampling. Robustness is quantified as the normalized area under the performance curve (AUC) over label-retention rates $p\in\{0.2,0.5,0.8,1.0\}$. Metrics include cell-type silhouette, modality-mixing score ($1-$Silhouette (omics)), and F1 silhouette. **(b)** Embedding quality as a function of annotation completeness. Line plots show median cell-type silhouette, modality-mixing score ($1-$Silhouette (omics)), and F1 silhouette across five runs with different random seeds at each downsampling level. Error bars indicate standard deviation. Statistical significance was assessed using a two-sided t-test.


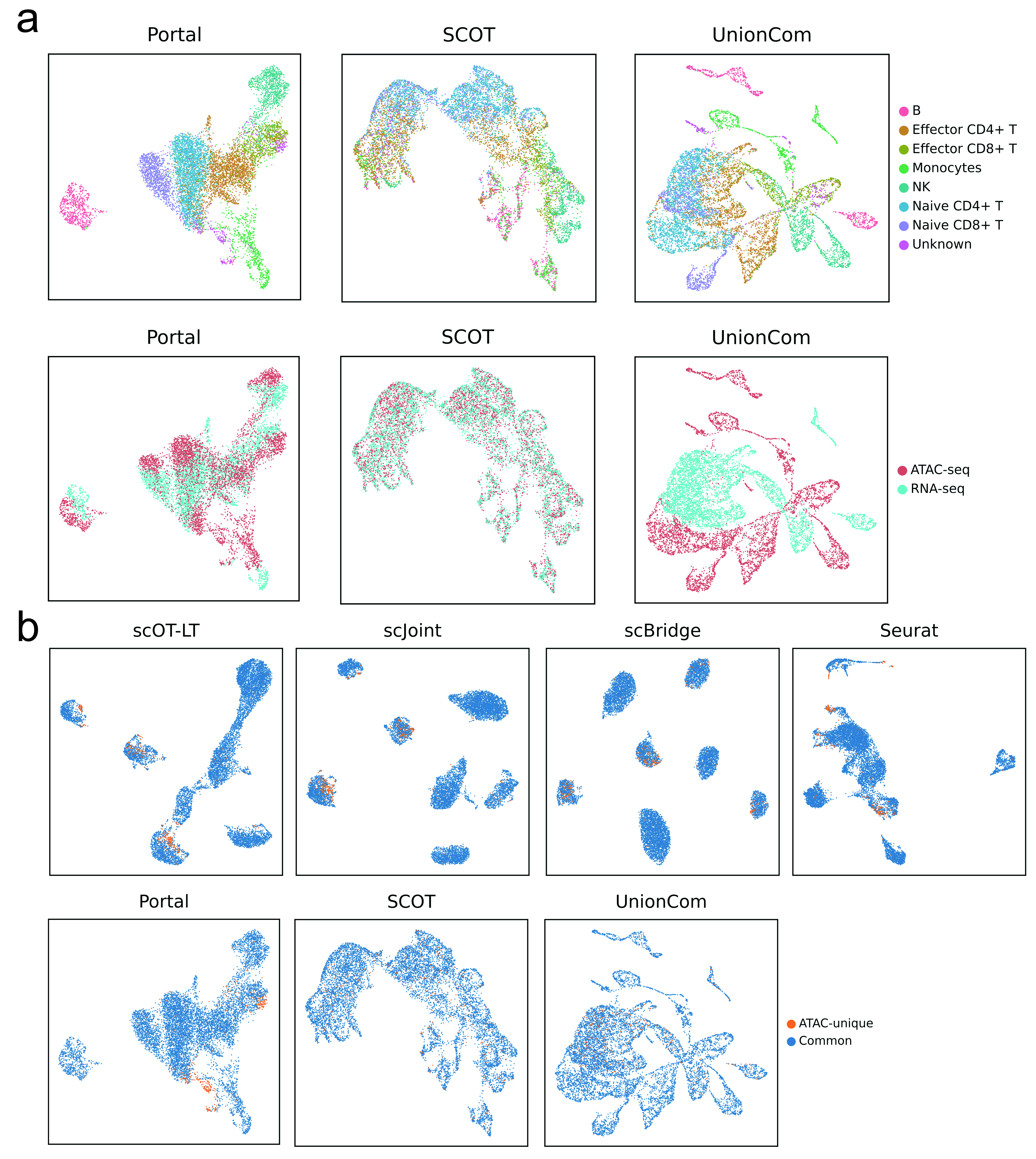


**Supplementary Figure S5.** Additional baseline comparisons for embeddings and novel-cell behavior in PBMC dataset integration. **(a)** UMAP visualizations for Portal, SCOT and UnionCom. Top row is colored by cell type; bottom row is colored by modality. **(b)** UMAP visualizations for scOT-LT and competing methods, colored by common cells and ATAC-unique cells.


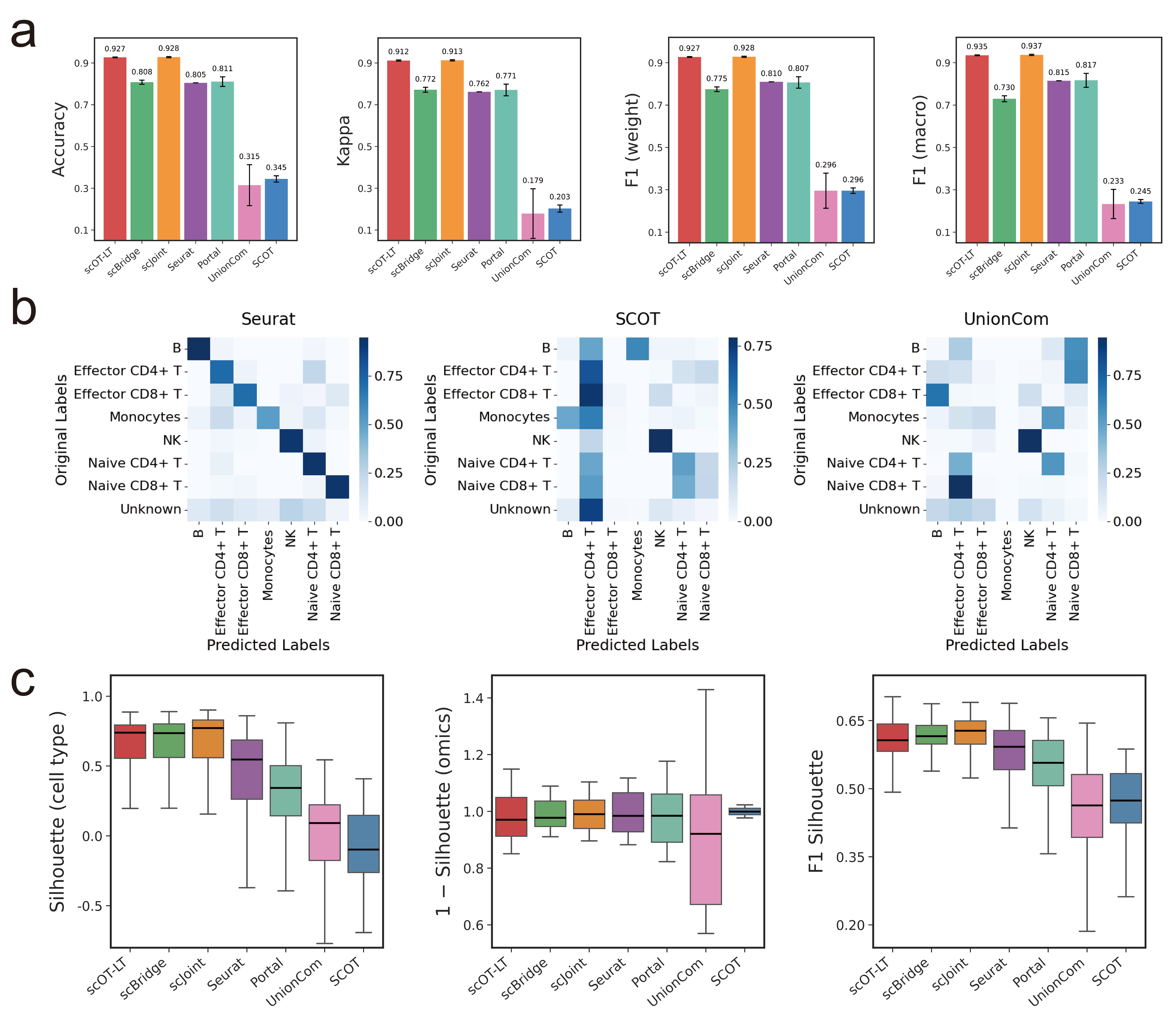


**Supplementary Figure S6.** Additional diagnostics of label transfer and embedding quality across methods on the PBMC dataset. **(a)** Label transfer performance across methods measured by accuracy, Cohen’s κ, weighted F1, and macro-F1. Values are summarized over five runs with different random seeds; error bars indicate standard deviation. Statistical significance was assessed using a two-sided t-test. **(b)** Confusion matrices for Seurat, SCOT and UnionCom. **(c)** Distribution of embedding-quality metrics across methods, including cell type silhouette, modality mixing score (1 − silhouette(omics)) and F1 silhouette.


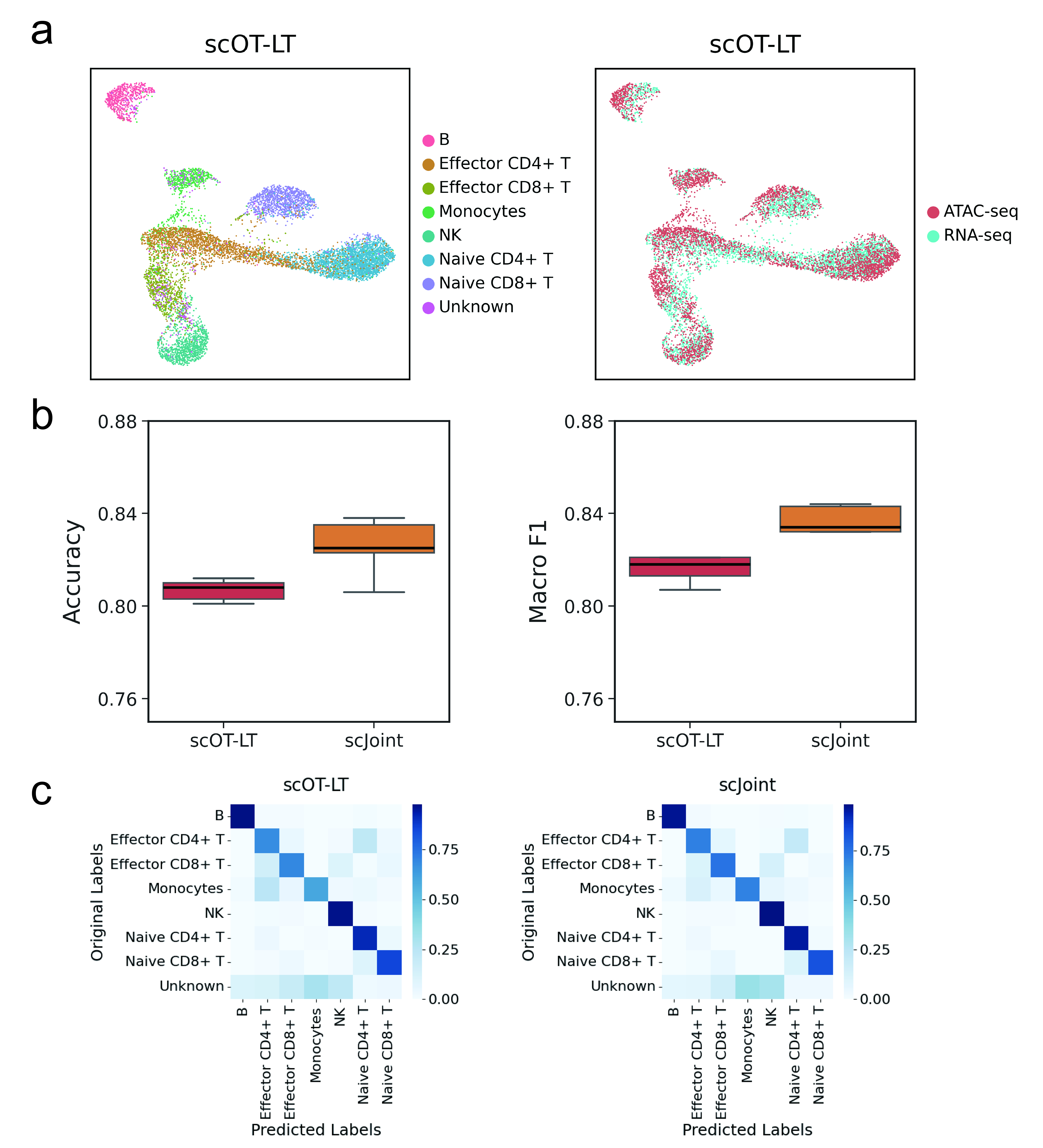


**Supplementary Figure S7.** PBMC ablation without protein measurements. **(a)** Joint UMAP embeddings generated by scOT-LT using only gene expression and gene-activity features. Left: cells colored by cell type. Right: cells colored by modality. **(b)** Label-transfer performance, summarized by accuracy and macro-F1 across five runs with different random seeds. **(c)** Confusion matrices comparing true and predicted labels for scOT-LT and scJoint.


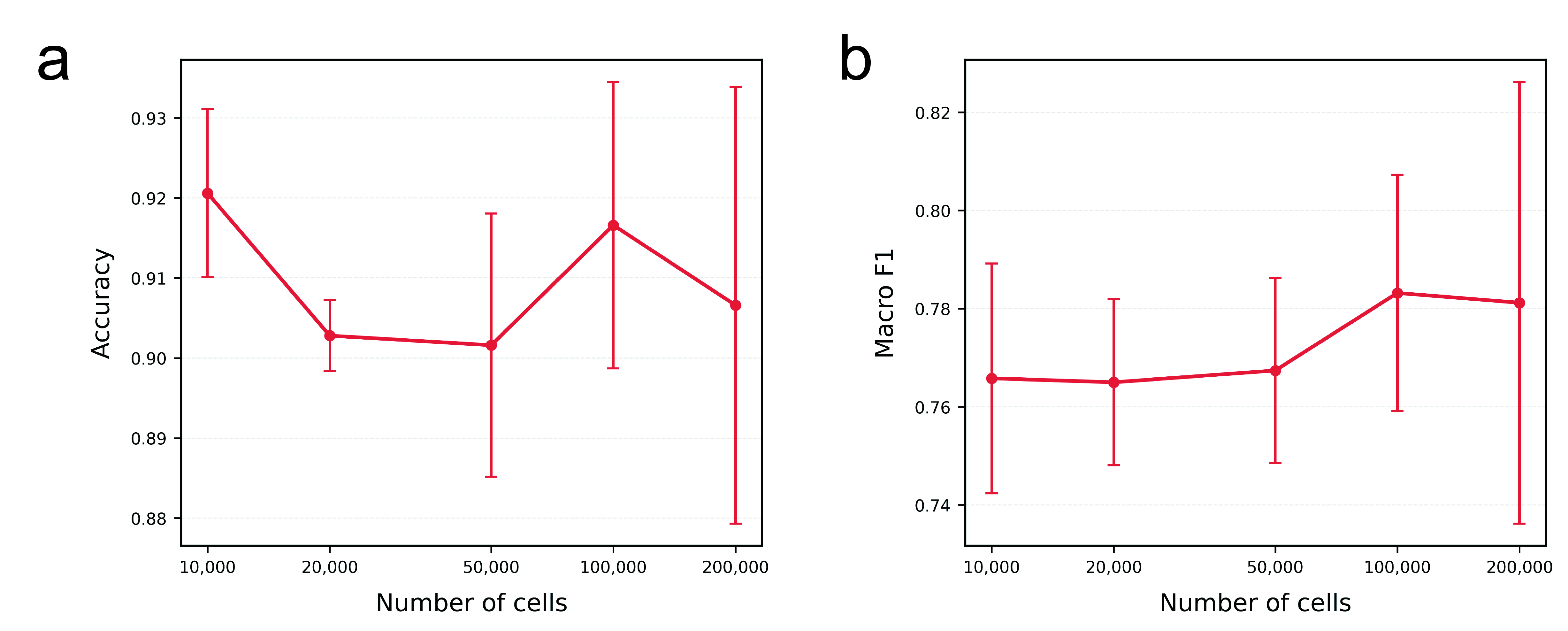


**Supplementary Figure S8.** Predictive performance of scOT-LT across increasing dataset sizes. Accuracy **(a)** and macro-F1 **(b)** are shown as mean ± SD over five independent runs, where five random subsets were generated and evaluated for each dataset size.

**Supporting Note**

**A: Evaluation metrics**

**Evaluation of label transfer effectiveness**

We evaluate the accuracy of label transfer by calculating the overall accuracy, weighted F1 score for cell-type classification, Adjusted Rand Index (ARI), and Normalized Mutual Information (NMI), based on a comparison between the true labels and predicted labels from scATAC-seq.

The overall accuracy is calculated considering only the common cell types between the scRNA-seq and scATAC-seq datasets. Specifically, let $\hat{y}_{i}$ and $y_{i}$ represent the predicted and true cell type annotations for cell $i$, respectively, $N_{\mathrm{com}}$ denote the subset of cells with common cell types in both the scRNA-seq and scATAC-seq datasets. The overall accuracy measures the proportion of correctly classified cells, as calculated by:

$$Accuracy= \frac{\sum_{i=1}^{N_{\mathrm{com}}} \delta(\hat{y}_{i}(y_{i}))}{N_{\mathrm{com}}}, \delta\left( a,b \right)=\left\{ \begin{aligned} 1 if a=b, \\ 0 otherwise. \end{aligned} \right.$$

The weighted F1 score for cell-type classification is the harmonic mean of precision and recall for each cell type:

$${F1}_{c}=2\frac{P_{c}\cdot R_{c}}{P_{c}+R_{c}},$$

$${F1}_{\mathrm{weighted}}=\frac{1}{N}\sum_{c=1}^{C} n_{c}\cdot{F1}_{c}$$

where $P_{c}$ and $R_{c}$ represent the precision and recall for class $c$, respectively, $C$ is the total number of cell types, $n_{c}$ is the number of cells in class $c$, and $N$ is the total number of cells.

Macro-averaged F1 Score is an alternative strategy for aggregating F1 scores in multi-class settings. Unlike the weighted F1 score, which weights each class by its sample size, the macro-averaged F1 assigns equal weight to each class by computing the arithmetic mean of the per-class F1 scores:

$$F1_{\text{macro}}=\frac{1}{C}\sum_{c=1}^{C} F1_{c}$$

where $F1_{c}$ is the F1 score for class $c$, and $C$ is the total number of classes. This metric treats every class equally and is therefore more sensitive to the model’s performance on rare or minority cell types, making it suitable for evaluating the overall balanced performance on imbalanced class distributions.

Cohen‘s Kappa (κ) measures the agreement between predicted labels and true labels, with the key advantage of accounting for the agreement expected by random chance (i.e., the probability of guessing correctly by accident). It provides a more robust measure of consistency than simple accuracy:

$$\kappa=\frac{p_{o}-p_{e}}{1-p_{e}}$$

where $p_{o}$ is the observed accuracy (overall accuracy), and $p_{e}$ is the expected agreement under random chance, calculated from the marginal distributions of the true and predicted labels. Kappa typically ranges from -1 to 1, with higher values indicating better agreement between predictions and true labels—agreement that surpasses random guessing. This metric is particularly useful for evaluating whether a classifier’s predictive capability is statistically significant in datasets with imbalanced cell type distributions.

**Evaluation of joint embedding performance**

To assess whether the joint embeddings generated by different methods demonstrate clustering structures that reflect biological signals or technical variations, we calculated the silhouette coefficient for each cell. Two types of groupings are analyzed: (1) grouping based on the modalities (scRNA-seq or scATAC-seq), referred to as the modality silhouette coefficient ($s_{\mathrm{modality}}$); (2) grouping based on known cell types, termed the cell-type silhouette coefficient ($s_{\mathrm{cellTypes}}$). In an optimal joint visualization, a low modality silhouette coefficient indicates that technical effects have been minimized, while a high cell-type silhouette coefficient suggests that cells are correctly clustered by cell type. The Euclidean distance for all methods is computed based on UMAP embedding.

Additionally, we calculated the harmonic mean of the two silhouette coefficients to derive the F1 score, defined as:

$${F1}_{\mathrm{sil}}=\frac{2\cdot(1-s_{\mathrm{modality}}^{'})\cdot s_{\mathrm{cellTypes}}^{'}}{1-s_{\mathrm{modality}}^{'}+s_{\mathrm{cellTypes}}^{'}}$$

where $s^{'}={(s+1)}/2$. A higher F1 score reflects better performance in aligning modalities and preserving biological signals.

**Evaluation of cell-to-cell pairing preservation**

FOSCTTM, which stands for “fraction of samples closer than the true match”, measures the preservation of cell-to-cell pairings in the neighborhood structure. This metric is calculated on two datasets with known cell-to-cell correspondence. The average FOSCTTM is calculated as follows:

$$\mathrm{FOSCTTM}_{\mathrm{avg}}=\frac{1}{2n}FOSCTTM,$$

$$FOSCTTM=\sum_{i=1}^{n} \frac{s^{i}}{n}+\sum_{i=1}^{n} \frac{t^{i}}{n},$$

$$s^{i}=\left| \left\{ j|d\left( z_{x_{j}},z_{y_{i}} \right)<d(z_{x_{i}},z_{y_{i}}) \right\} \right|,$$

$$t^{i}=\left| \left\{ j|d\left( z_{x_{i}},z_{y_{j}} \right)<d(z_{x_{i}},z_{y_{i}}) \right\} \right|.$$

where $n$ is the number of cells in datasets $X$ and $Y$, and $z_{x_{i}}$ and $z_{y_{i}}$ are paired cells. The value $s^{i}$ and $t^{i}$ represent the number of cells in dataset $X$ and $Y$, respectively, that are closer to the cell $i$ in one dataset than to its true match in another dataset. The average FOSCTTM ranges from 0 to 1, with lower values indicating higher accuracy in preserving the matching relationships.

**Evaluation of robustness**

We employed the area under the curve (AUC) as an overall performance evaluation metric. For a performance curve $y=f(x)$, the AUC is defined as the integral of the curve over the interval $[a,b]$:

$$AUC=\int_{a}^{b} f(x)\text{ }dx.$$

Specifically, we set the horizontal axis as the proportion parameter $p$ and the vertical axis as the median performance $m(p)$ at that proportion, and calculated the median AUC:

$$\mathrm{AU}C_{\text{med}}=\int_{0.2}^{1.0} m(p)\text{ }dp,$$

where $p$ takes values at 0.2, 0.5, 0.8, and 1.0. In practice, the integral was approximated using the trapezoidal rule:

$$\mathrm{AUC}_{\mathrm{med}}=\sum_{i} \frac{m\left( p_{i} \right)+m\left( p_{i+1} \right)}{2}(p_{i+1}-p_{i}).$$

For better comparability, we normalized $\mathrm{AU}C_{\text{med}}$ into an "average performance":

$$\mathrm{AU}C_{\text{med}}^{\text{norm}}=\frac{\mathrm{AU}C_{\text{med}}}{1.0-0.2}.$$

This metric comprehensively reflects the robustness of the method across different proportions.

**Entropy and enrichment**

To assess how well computational approaches identify cell types that are exclusive to scATAC-seq, we adopted two evaluation measures originally introduced in scGCN: normalized entropy and enrichment score [1]. The normalized entropy (NE) is formulated as

$$NE=\frac{1}{M{log}_{2}|C_{R}|}\sum_{i} \sum_{j\in C_{R}} \frac{S_{i,j}}{\sum_{j\in C_{R}} S_{i,j}}{log}_{2}\frac{S_{i,j}}{\sum_{j\in C_{R}} S_{i,j}},$$

where $S_{i,j}=\frac{P_{i,j}}{Q_{j}}$, $P_{i,j}$ denotes the predicted probability that a cell $i$ from a scATAC-seq-specific cell type label is assigned to the scRNA-seq cell type $j$, whereas $Q_{j}$ corresponds to the prevalence of cell type $j$ in the scRNA‑seq data, serving as a background probability. The set $C_{R}$ contains all cell types present in the scRNA‑seq reference, and $M$ indicates the total number of scATAC‑seq cells carrying unique cell‑type labels. Division by $\log_{2}\mid C_{R}\mid$ ensures that the resulting score lies within the interval [0, 1]. The second metric, the enrichment score (ES), is computed as

$$ES=\frac{1}{M}\sum_{i} {max}_{j\in C_{R}}\frac{S_{i,j}}{\sum_{j\in C_{R}} S_{i,j}}$$

Similarly confined between 0 and 1, this score quantifies the tendency of predictions to concentrate on a single cell type. For cell types observed only in the scATAC‑seq assay, an effective method should yield a high normalized entropy together with a low enrichment score. To integrate both aspects into a single performance indicator, we also derived a combined F1 score

$$F_{1}=2\frac{NE\cdot(1-ES)}{NE+(1-ES)}$$

**Statistical analysis**

Statistical analyses were performed using the SciPy Python package [2]. For all main quantitative experiments, each method was evaluated over five runs with different random seeds and model initializations. Reported results were summarized across runs, and statistical significance between scOT-LT and competing methods was assessed using a two-sided t-test, with $p-value<0.05$ considered statistically significant.

**B: Settings for comparing methods**

***scJoint:*** For scJoint [3], we employed the official code from https://github.com/SydneyBioX/scJoint and applied the hyperparameters provided in the code example or recommended in the paper. Since scJoint binarizes the gene expression and gene activity matrices as an initial step, no further data preprocessing is necessary. The algorithm directly generates embeddings for both scRNA-seq and scATAC-seq data (_embeddings.txt) and the label transfer results for scATAC-seq data (_knn_predictions.txt).

***scBridge:*** scBridge [4] employs a semi-supervised framework to combine scRNA‑seq and scATAC‑seq data. First, it extracts distinctive cell‑type signatures, termed prototypes, from labeled scRNA‑seq references. It then processes scATAC‑seq profiles as estimates of gene activity and selects, for integration, those scATAC‑seq cells that exhibit strong alignment between their chromatin accessibility patterns and the expression profiles of the prototypes. The analysis was performed using the standard parameters provided in the official repository <https://github.com/XLearning-SCU/scBridge/tree/main>.

***Seurat:*** All datasets were analyzed using the Seurat v5 R package [5]. The input data included the raw count matrix for scRNA-seq and the unnormalized gene activity score matrix for scATAC-seq, both of which were subsequently normalized using the NormalizeData function in Seurat. For the CITE-seq and ASAP-seq datasets, the input consisted of concatenated, log-transformed matrices of normalized gene expression and gene activity, along with log-transformed ADT matrices. The FindVariableFeatures function with the vst method was applied to select the 2000 most variable genes from the scRNA-seq dataset. The FindTransferAnchors function was then used to identify anchors between scRNA-seq and scATAC-seq datasets, with 'cca' chosen for dimensionality reduction. The TransferAnchors function was employed to impute the scATAC-seq data, with anchors weighted based on the latent semantic indexing of scATAC-seq dimensions. Finally, PCA was performed on the merged scRNA-seq and imputed scATAC-seq data matrices, and joint visualization was conducted using the first 30 principal components.

***Portal:*** The portal-sc Python package [6] (v1.0.4) was used to analyze the data. Following the default pipeline, the gene expression and gene activity matrices were preprocessed using the model.preprocess function. Data integration was then performed with the model.train function, setting the training steps (training_steps) to 3000 for all datasets. The integration results were obtained by running the model.eval function, extracting the outputs from model.latent. Since Portal does not support label transfer, the KNeighborsClassifier function (k=30) from the scikit-learn Python package [7] (v0.24.1) was used to transfer cell annotations from scRNA-seq data to scATAC-seq data.

***SCOT:*** SCOT [8] (v1.0) was downloaded from https://github.com/rsinglab/SCOT for integrating scATAC-seq and scRNA-seq data. We tested multiple data preprocessing strategies and selected the configuration that yielded the best performance. Specifically, for the BMMC and MultiMAP datasets, dimensionality reduction with PCA was applied. For the PBMC dataset, log1p transformation followed by StandardScaler was used for normalization.

***UnionCom:*** The Python package unioncom [9] (v0.4.0) was used for integrating scATAC-seq and scRNA-seq data. For UnionCom, various preprocessing methods were systematically compared, and the most effective protocol was adopted. No additional preprocessing was performed on the BMMC dataset. For the MultiMAP and PBMC datasets, scRNA‑seq data underwent log1p transformation, while scATAC‑seq data were processed using TF‑IDF transformation followed by L2 normalization and PCA for dimensionality reduction.

**Reference**

1. Song QQ, Su J, Zhang W. scGCN is a graph convolutional networks algorithm for knowledge transfer in single cell omics, Nature Communications 2021;12:11.

2. Virtanen P, Gommers R, Oliphant TE et al. SciPy 1.0: fundamental algorithms for scientific computing in Python, Nature Methods 2020;17:261-272.

3. Lin YX, Wu TY, Wan S et al. scJoint integrates atlas-scale single-cell RNA-seq and ATAC-seq data with transfer learning, Nature Biotechnology 2022;40:703-710.

4. Li YF, Zhang D, Yang MX et al. scBridge embraces cell heterogeneity in single-cell RNA-seq and ATAC-seq data integration, Nature Communications 2023;14:14.

5. Hao YH, Stuart T, Kowalski MH et al. Dictionary learning for integrative, multimodal and scalable single-cell analysis, Nature Biotechnology 2024;42:22.

6. Zhao J, Wang GF, Ming JS et al. Adversarial domain translation networks for integrating large-scale atlas-level single-cell datasets, Nature Computational Science 2022;2:317-330.

7. Pedregosa F, Varoquaux G, Gramfort A et al. Scikit-learn: Machine Learning in Python, Journal of Machine Learning Research 2011;12:2825-2830.

8. Demetci P, Santorella R, Sandstede B et al. SCOT: Single-Cell Multi-Omics Alignment with Optimal Transport, Journal of Computational Biology 2022;29:3-18.

9. Cao K, Bai XQ, Hong YG et al. Unsupervised topological alignment for single-cell multi-omics integration, Bioinformatics 2020;36:48-56.
